# Lipidomic Evidence for Cannabidiol-Induced Remodeling of Cellular Lipid Composition in SH-SY5Y Cells

**DOI:** 10.3390/cells15181714

**Published:** 2026-09-21

**Authors:** Oliver Walzer, Vincent Konrad Johannes Erhardt, Jill Sven René Zügner, Natalya V. Izarova, Kristina Endres, Heike Sabine Grimm, Tobias Hartmann, Marcus Otto Walter Grimm

**Affiliations:** 1 Experimental Neurology, Saarland University, 66421 Homburg, Germany; oliver.walzer@uni-saarland.de (O.W.); vincent.erhardt@uni-saarland.de (V.K.J.E.); jizu00001@uni-saarland.de (J.S.R.Z.); natalya.izarova@uni-saarland.de (N.V.I.); heike.grimm@srh.de (H.S.G.); tobias.hartmann@uks.eu (T.H.); 2 Faculty of Computer Sciences and Microsystems Technology Kaiserslautern, University of Applied Sciences, 66482 Zweibrücken, Germany; kristina.endres@hs-kl.de; 3 Department of Psychiatry and Psychotherapy, University Medical Center, Johannes Gutenberg University Mainz, 55122 Mainz, Germany; 4 Nutrition Therapy and Counseling, Campus Rheinland, SRH University of Applied Health Sciences, 51377 Leverkusen, Germany; 5 Deutsches Institut für Demenzprävention (DIDP), Saarland University, 66421 Homburg, Germany

**Keywords:** cannabidiol (CBD), lipidomics, neurodegeneration, phospholipid remodeling, neuronal lipid metabolism

## Abstract

Cannabidiol (CBD) is increasingly discussed in relation to neurodegenerative disorders, including Alzheimer’s disease (AD), yet its effects on cellular lipid profiles remain poorly understood. Using targeted lipidomics, we investigated whether CBD alters the lipidome of SH-SY5Y wildtype (wt) cells and whether the principal phosphatidylcholine response is also observed in cells expressing amyloid precursor protein with the Swedish mutation (APPswe). CBD induced a broad, class-specific lipid response. Total diacyl and ether-linked phosphatidylcholines increased. Within these classes, many species with lower degrees of unsaturation increased, whereas several highly polyunsaturated ether-linked phosphatidylcholines declined. Lysophosphatidylcholines were broadly reduced, total sphingomyelin abundance declined, free carnitine decreased, and most measured acylcarnitine species also declined. Together, in wt cells, these changes are consistent with a relative shift toward a less polyunsaturated lipid profile. CBD-associated changes in both phosphatidylcholine classes were also observed in stably APPswe-expressing SH-SY5Y cells. Because the affected lipid classes have been implicated in AD-related lipid dysregulation, these findings link CBD exposure to an AD-relevant lipid context and provide a rationale for investigating the functional consequences of this response in more complex neurodegeneration-relevant models.

## 1. Introduction

Cannabidiol (CBD) is a non-psychoactive phytocannabinoid naturally occurring in Cannabis sativa. Structurally, it is related to Δ^9^-tetrahydrocannabinol (Δ^9^-THC), but CBD lacks the closed oxygen-containing pyran ring present in Δ^9^-THC [1]. CBD is lipophilic, has low oral bioavailability, and exhibits high plasma protein binding [2]. Consequently, it penetrates the blood–brain barrier (BBB) and distributes into fatty tissue and organs [3]. Reported half-life after repeated dosing ranges from 56 h to more than 134 h in some human studies, while single-dose administration or alternative application routes result in shorter half-lives [4]. Animal studies indicate brain-to-plasma ratios of approximately 40–60% following systemic administration, with BBB penetration increasing over time and in a dose-dependent manner [3].

A wide variety of CBD products is available, including drops, soft gels, buccal sprays, and combined formulations. Many of these products are not registered as medicinal products and may have uncertain purity [5].

In recent years, interest has increased in CBD as a potential therapeutic agent for a wide range of conditions, while its use as a lifestyle product has also prompted stricter regulation of distribution and advertising in many countries [6]. In clinical practice, purified CBD is available as Epidyolex for the adjunctive treatment of rare epileptic syndromes, including Lennox–Gastaut and Dravet syndromes, and CBD is a component of nabiximols (Sativex), which is used to treat spasticity in multiple sclerosis [7,8].

Off-label use of CBD includes conditions such as anxiety and sleep disorders [9]. CBD has also been investigated as an adjunct in analgesic therapy, although formulations containing psychoactive Δ^9^-THC may be more effective in severe pain conditions [10].

The standard dosage of CBD varies depending on the underlying cause of treatment. The dosage for Epidyolex is relatively high, ranging from 20 mg/d up to 1500 mg/d in different studies and for different indications [5]. Among the reported side effects are somnolence, fatigue, decreased appetite, and diarrhea [1]. Overall, CBD is generally well tolerated [11]. When co-administered with drugs metabolized by the cytochrome P450 system, interactions may occur, and in rare cases, elevation of liver enzymes have been reported [1,12].

A toxic dosage is not well-defined in humans, whereas in vitro cytotoxicity can be observed in a dose-dependent manner, depending on the respective cell type [13,14].

Rather than acting as a potent direct agonist, CBD can be understood as a modulator of endocannabinoid-system tone. This system primarily comprises CB_1_ and CB_2_ receptors and the major endocannabinoids anandamide and 2-arachidonoylglycerol [15]. CBD has low affinity for CB_1_ and CB_2_ receptors [16] and acts as a negative allosteric modulator of CB_1_ [17]. It may also indirectly increase anandamide concentrations by inhibiting fatty-acid amide hydrolase [18].

CBD interacts with several molecular targets, including 5-HT1A receptors, transient receptor potential channels, and PPARγ [19]. PPARγ regulates multiple aspects of lipid metabolism, and PPARγ-dependent regulation of SCD1 expression has been reported in different experimental systems [20]. Moreover, CBD-associated PPARγ signaling has been described in other cell types [21,22,23]. These observations provide a literature-based rationale for considering PPARγ- and SCD1-related pathways as possible contributors to CBD-associated lipid remodeling. Their involvement in the present cellular system, however, requires direct experimental investigation.

CBD has been widely discussed in relation to Alzheimer’s disease (AD), although clinical evidence in this context remains limited [24]. Across randomized clinical trials of CBD conducted for other indications, adverse effects have been reported [25], whereas a preclinical study in the 5xFAD model reported reduced amyloid-β pathology and improved cognitive outcomes [26].

Preclinical studies have also associated CBD exposure with anti-inflammatory and neuronal signaling pathways relevant to neurodegenerative disorders [24,27,28,29]. Together, these observations provide a rationale for further investigation while requiring careful distinction between findings in specific experimental systems and clinical conclusions.

The integrity and functionality of human cells, particularly neuronal cells, depend substantially on compartmentalization by lipid bilayers. Changes in lipid composition have been implicated in the pathophysiology of AD [30,31,32].

Against this background, we investigated whether CBD alters the cellular lipid profile of undifferentiated human neuroblastoma-derived SH-SY5Y cells using targeted lipidomics. The 1 µM/72 h experiment characterized the lipid response under sustained exposure, while complementary analyses examined the central phosphatidylcholine response at a lower CBD concentration, after a shorter exposure period, and in stably APPswe-expressing SH-SY5Y cells. The study was designed to characterize CBD-associated lipid remodeling and to generate testable hypotheses concerning its possible relevance to AD-related lipid dysregulation.

## 2. Materials and Methods

### 2.1. Chemicals, Reagents, Standards

Unless otherwise stated, all chemicals, reagents, and standards were purchased from Thermo Fisher Scientific Inc. (Waltham, MA, USA).

### 2.2. Drug Preparation

CBD with a purity of ≥99% (HPLC) was purchased from PhytoLab GmbH & Co. KG (Vestenbergsgreuth, Germany) and dissolved in HPLC-grade ethanol at a stock concentration of 100 mM. Before the 1 µM/72 h lipidomic experiment, SH-SY5Y wt cells were exposed to 0.25, 0.5, 1.0, or 2.5 µM CBD for 72 h. LDH release did not indicate an increased loss of plasma-membrane integrity at the concentrations tested (Supplementary Figure S1). Within this concentration range, 1 µM CBD was selected as a predefined low-micromolar exposure condition for investigating sustained cellular lipid remodeling over 72 h. Low-micromolar CBD exposure, including 1 µM, has also been used in previous SH-SY5Y-based studies [33,34]. To extend the central phosphatidylcholine findings, wt cells were additionally exposed to 0.25 µM CBD for 24 or 72 h and to 1 µM CBD for 24 h. For comparison across conditions, the 1 µM/72 h dataset served as the reference condition.

### 2.3. Cell Culture

Human neuroblastoma-derived SH-SY5Y wt cells were examined in an undifferentiated state. All cryocultures used had been tested for the absence of mycoplasma using a PCR-based test kit (Pre-aliquoted Mycoplasma Detection Kit for conventional PCR from Minerva biolabs, Skillman, NJ, USA). Cells were cultured at 37 °C in a humidified atmosphere containing 5% CO_2_ in Dulbecco’s modified Eagle’s medium supplemented with 10% fetal bovine serum (FBS) and 1% non-essential amino acids. Cells were seeded into individual cell culture dishes in P3 after thawing. With a confluence of the seeded cells of approximately 80%, the culture dishes were randomized to the different incubation conditions, and the FBS content of the medium was reduced from 10 to 2.5%. After a further 16 h, exposure was initiated by replacing the culture medium with fresh treatment medium containing 2.5% FBS and CBD at the respective concentration or the corresponding solvent control. The serum-reduced condition was selected to reduce the contribution of serum-derived lipids while maintaining sufficient serum support to minimize nonspecific cellular stress and potential cytotoxicity associated with more extensive serum deprivation or complete serum withdrawal. CBD-treated and solvent-control cells received the same FBS concentration and were subjected to identical medium-change schedules.

SH-SY5Y cells stably transfected with pCEP4-APPswedish and expressing human APP695 carrying the Swedish mutation (APPswe) were cultured as described previously [35,36]. These cells were maintained under selection with 600 µg/mL hygromycin B, including during the experimental exposure period, and were likewise examined in an undifferentiated state. WT cells were exposed to 0.25 µM CBD for 24 or 72 h or to 1 µM CBD for 24 or 72 h. APPswe-expressing cells were exposed to 1 µM CBD for 72 h. During each 72 h exposure, the treatment medium was renewed after 24 and 48 h, each time using fresh treatment medium containing 2.5% FBS and CBD at the respective concentration or the corresponding solvent control. Cell morphology was monitored qualitatively by light microscopy throughout the exposure period.

### 2.4. Assessment of Plasma-Membrane Integrity and Cellular Biomass

CBD-associated effects on plasma-membrane integrity were assessed by measuring LDH release into the culture medium using the Cytotoxicity Detection Kit (Roche Diagnostics GmbH, Mannheim, Germany) according to the manufacturer’s instructions, as described previously [35,37]. The preliminary concentration screen was performed in 6-well plates and is presented in Supplementary Figure S1. Total cellular protein content was determined after the 72 h exposure period using the Pierce BCA Protein Assay Kit (Thermo Fisher Scientific Inc.) according to the manufacturer’s instructions [35,38]. Total protein served as a measure of overall cellular biomass and was also used for subsequent sample normalization. Comparable protein concentrations in CBD-treated and solvent-control samples were consistent with comparable overall cellular biomass and did not indicate a major difference in cell accumulation under the experimental conditions (Supplementary Figure S2). Cell morphology was additionally monitored qualitatively by light microscopy throughout the exposure period. BCA measurements were performed in technical triplicate.

### 2.5. Analysis of Lipidome

#### 2.5.1. Sample Preparation

Incubation was performed in 10 cm cell-culture dishes. At the end of the exposure period, the culture medium was removed, and the cells were washed twice by adding and immediately removing HPLC-grade water; each washing step lasted only a few seconds. This rapid terminal washing procedure was used to remove residual culture medium, serum-derived components, and salts that could contribute to matrix effects and ion suppression during mass-spectrometric analysis, consistent with related adherent-cell metabolomics workflows [39,40]. No cell detachment or apparent morphological alterations were observed during these brief washing steps. Immediately thereafter, the cells were harvested in 180 µL HPLC-grade water, and the complete harvested suspension was retained and mechanically lysed. CBD-treated and solvent-control samples underwent identical washing and harvesting procedures. Cells were lysed mechanically with seven ceramic beads in microcentrifuge tubes from VWR (Radnor, PA, USA) using a MiniLys from PEQLAB (Erlangen, Germany) for 30 s at the maximum setting. Protein content was subsequently determined by the BCA assay, and CBD-treated samples together with the corresponding solvent control samples were adjusted to a uniform protein content with HPLC-grade water. Protein concentrations did not differ markedly between the CBD-treated and solvent-control samples (Supplementary Figure S2).

Cell lipids were isolated by solid–liquid extraction, as described in detail in Grimm et al. and Lauer et al. with the following main steps [41,42]: a filter plate (0.45 µm, Merck, Darmstadt, Germany) was fixed on a deep well plate and Whatman paper of 6 mm diameter was placed in the chambers. A volume of 10 µL of protein-adjusted sample was mixed with 6 µL of the internal-standard mixture at the concentrations specified in Supplementary Table S1 and transferred to the Whatman paper. The internal standards were added before lipid extraction and were used to normalize variability associated with sample extraction and instrumental response. The Whatman paper was dried for 45 min under nitrogen flow. Then, 20 µL phenylisothiocyanate solution, prepared from phenylisothiocyanate (Merck)/ethanol/water/pyridine (Merck) (3:19:19:19, *v*/*v*/*v*/*v*), was added, the plate was incubated for 20 min at room temperature, and a second drying under N_2_ was performed. Ammonium acetate solution (5 mM in methanol) was added, and the plate was incubated on a shaker for 30 min at 450 rpm. After centrifugation for 2 min at 500 g, the filter plate was removed, and the extracts were diluted with 600 µL running buffer, containing 5 mM ammonium acetate in a mixture of water/methanol (97:3, *v*/*v*).

Each cell-culture dish constituted one independently treated and processed biological replicate and served as the experimental unit for statistical analysis. Every dish was independently incubated with CBD or the corresponding solvent control, harvested, subjected to lipid extraction, and analyzed as a separate sample by mass spectrometry. No cell samples or lipid extracts were pooled at any stage. Each lipid extract was measured in three technical mass-spectrometric replicates, which were averaged before statistical analysis. Thus, each cell-culture dish contributed one biological value to the statistical analysis. The exact biological sample sizes for each experimental condition and the number and allocation of independent culture runs are reported in Supplementary Table S8.

#### 2.5.2. Mass Spectrometry

An Orbitrap ID-X mass spectrometer (Thermo Fisher Scientific Inc.) in conjunction with an autosampler unit of the Vanquish neo system (Thermo Fisher Scientific Inc.) and a 4000-quadrupole linear-ion trap (QTrap) with a Turbo Spray ion source (AB Sciex, Darmstadt, Germany) in conjunction with an autosampler unit of the Agilent HPLC 1200 series (Santa Clara, CA, USA) were used to perform the lipid analysis. Positive-ion mode was used for a targeted panel comprising signals assigned to diacyl phosphatidylcholine sum species (PCaa), the assay-specific PCae category, lysophosphatidylcholines (LPC), sphingomyelins (SM), free carnitine, and acylcarnitines. Both platforms were used for the targeted analysis of the five measured analyte groups; neither platform was dedicated to a particular lipid class. The same targeted analyte panel and designated internal standards were used on both platforms. The reported complementary phosphatidylcholine datasets comprised SH-SY5Y wt cells exposed to 0.25 µM CBD for 24 or 72 h or to 1 µM CBD for 24 h and stably APPswe-expressing SH-SY5Y cells exposed to 1 µM CBD for 72 h. These datasets were acquired using the Orbitrap ID-X. Measurement data were processed using TraceFinder 5.2 (Thermo Fisher Scientific Inc.) and Analyst 1.4.2 (AB Sciex) to obtain signal intensities for the monitored precursor/product ion pairs. The measurement conditions and monitored transitions are provided in Supplementary Tables S2–S5.

PCaa denotes phosphatidylcholines assigned as containing two ester-linked chains. PCae is retained as the operational analytical designation for nominally ether-containing phosphatidylcholine sum species. The analytical workflow did not distinguish plasmanyl from plasmenyl species, and for specified transitions, isobaric highly unsaturated diacyl phosphatidylcholine compositions cannot be excluded, as indicated in Supplementary Tables S4 and S5. The PCae signals are therefore not interpreted as structurally confirmed plasmalogens. In the notation PC x:y, x represents the total number of carbon atoms and y the total number of double-bond equivalents across the lipid chains. Individual fatty-acyl chains, their sn-positions, and double-bond positions were not resolved. LPC species contain one fatty-acyl chain, but their double-bond positions were likewise not determined [43].

Analytical consistency across individual samples was monitored through the responses of the designated internal standards added before extraction; their signal intensities were inspected for comparability within each analytical sequence. Each extract was analyzed in technical triplicate in three successive passes through the respective sample set. The three normalized measurements were averaged before statistical analysis and were not treated as independent biological observations.

The internal-standard mixture contained 06:0 PC (DHPC; 3.33 µM) for PCaa and PCae, 19:0 Lyso PC (3.33 µM) for LPC, 06:0 SM (d18:1/6:0; 4.72 µM) for SM, and octanoyl-L-carnitine-d3 (16.64 µM) and palmitoyl-L-carnitine-d3 (18.21 µM) for free carnitine and acylcarnitines. Carnitine-related signals were normalized to the arithmetic mean of the peak areas of the two deuterated carnitine standards. Internal-standard detection limits based on linear calibration curve and blank measurements were 13 ± 6 nM for 06:0 PC (DHPC), 18 ± 5 nM for 19:0 Lyso PC, 23 ± 13 nM for 06:0 SM (d18:1/6:0), 67 ± 12 nM for octanoyl-L-carnitine-d3, and 136 ± 31 nM for palmitoyl-L-carnitine-d3. For each standard, 14 concentration levels were prepared and 18 blank measurements were performed; the determination was conducted in three parallel extraction series, and values are reported as mean ± SD in Supplementary Table S1.

The analyzed panel covered complementary aspects of cellular lipid biology. PCaa, PCae, and LPC provide information on phosphatidylcholine composition and remodeling, sphingomyelins represent an important sphingolipid component of cellular membranes, and carnitine and acylcarnitine abundances provide information on cellular acyl-group handling. Alterations in these groups have previously been implicated in AD-related lipid dysregulation [30,31,32]. The distribution of the analyzed subclasses is summarized in Supplementary Table S6.

#### 2.5.3. Statistical Analysis

For each lipid extract, the three technical mass-spectrometric measurements were normalized and averaged before statistical analysis, and each independently treated and processed cell-culture dish contributed one biological value. PCaa and PCae signals were normalized to 06:0 PC (DHPC), LPC signals to 19:0 Lyso PC, and SM signals to 06:0 SM (d18:1/6:0). Free-carnitine and acylcarnitine signals were normalized to the arithmetic mean of the peak areas of octanoyl-L-carnitine-d3 and palmitoyl-L-carnitine-d3 (Supplementary Table S1).

Within each analytical run and incubation condition, the mean and standard deviation of each lipid species were calculated once using all biological samples. A biological sample was excluded from the analysis of a particular lipid class when more than two thirds of the measured species within that class fell outside the corresponding mean ± 1 SD interval. The criterion was applied separately to each lipid class and in a fixed, non-iterative manner: all sample flags were determined from the initial complete dataset before any value was removed. This class-wide quality criterion was used to identify coordinated atypical profiles that could arise from analytical variation during extraction, sample transfer or reconstitution, variation in introduced sample volume, transient disturbances of sample introduction, matrix-dependent ion suppression or enhancement, variation in spray or ionization efficiency, or transient changes in instrumental response. Recognition of atypical sample profiles and control of unwanted analytical variation are established components of mass-spectrometry-based lipidomics and metabolomics workflows [44,45,46,47,48].

**Figure 1:**
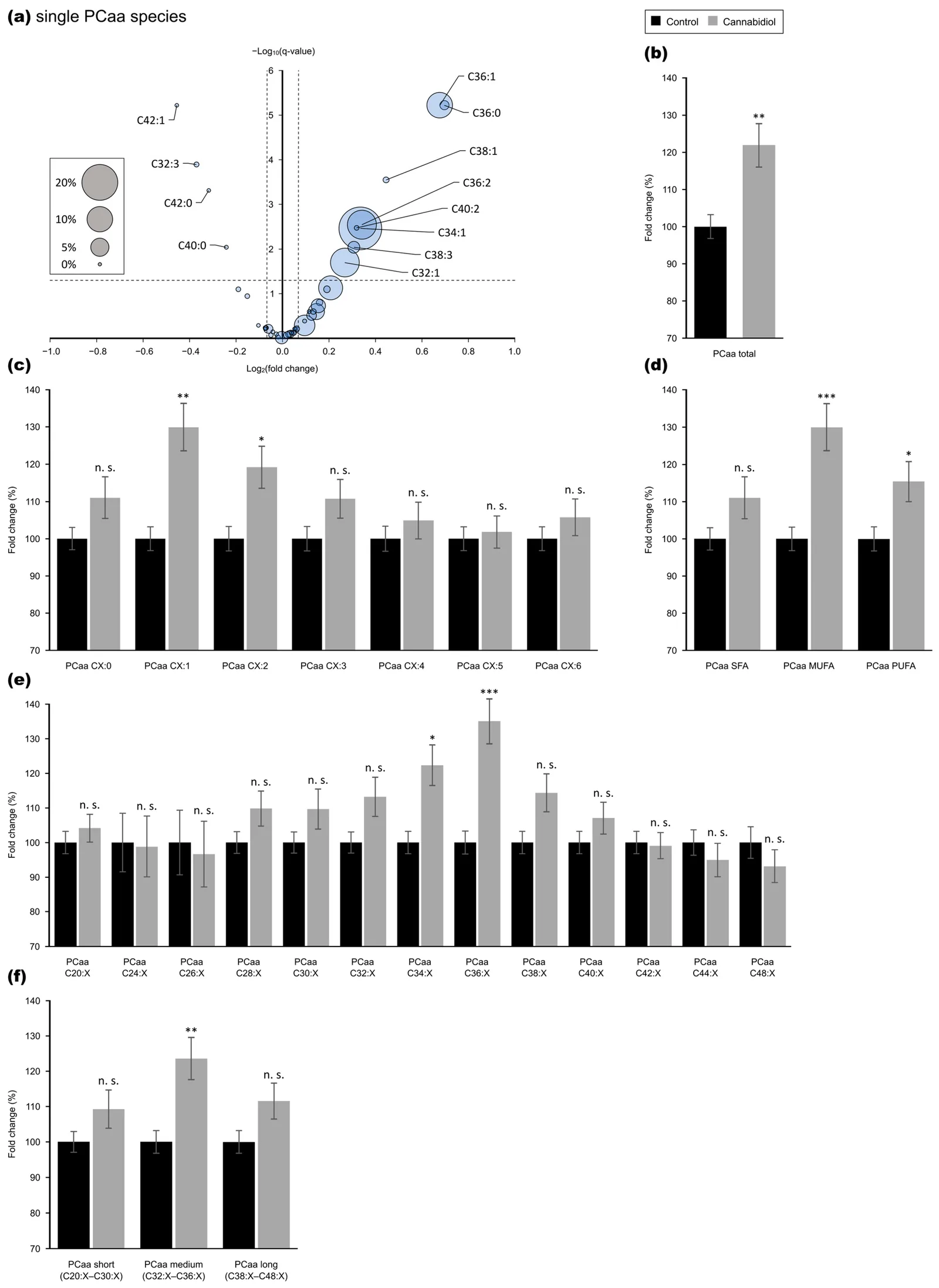
Diacyl-phosphatidylcholine (PCaa) levels in homogenates of SH-SY5Y wt cells incubated with 1 µM CBD for 72 h compared to the solvent control. (**a**) Fold changes of the individual PCaa species are shown as a volcano plot. The bubble area represents the proportion of each species in the PCaa lipid class in the CBD-treated group. Gray bubbles show reference proportions. The vertical dashed lines represent the mean standard error of the mean (mean SEM), and the horizontal line marks q = 0.05. Significantly changed species are named. (**b**–**f**) Bar charts showing the relative fold change of the PCaa total (**b**), the relative fold changes of PCaa species grouped by their saturation (**c**) or grouped by classes of saturation (**d**) and the relative fold changes of PCaa species grouped by their chain length (**e**) or grouped by classes of chain length (**f**). Error bars represent the SEM. Significance classifications are based on BH-adjusted *p*-values (q): * q ≤ 0.05, ** q ≤ 0.01, and *** q ≤ 0.001; n. s. = non-significant. For the class total in panel (**b**), the same thresholds refer to the Welch’s *t*-test *p*-value. The statistical analyses applied are described in detail in Section 2.5.3. The corresponding Welch’s *t*-test and BH-adjusted *p*-values are provided in Supplementary Table S7. The underlying n-counts are listed in Supplementary Table S8.

**Figure 2:**
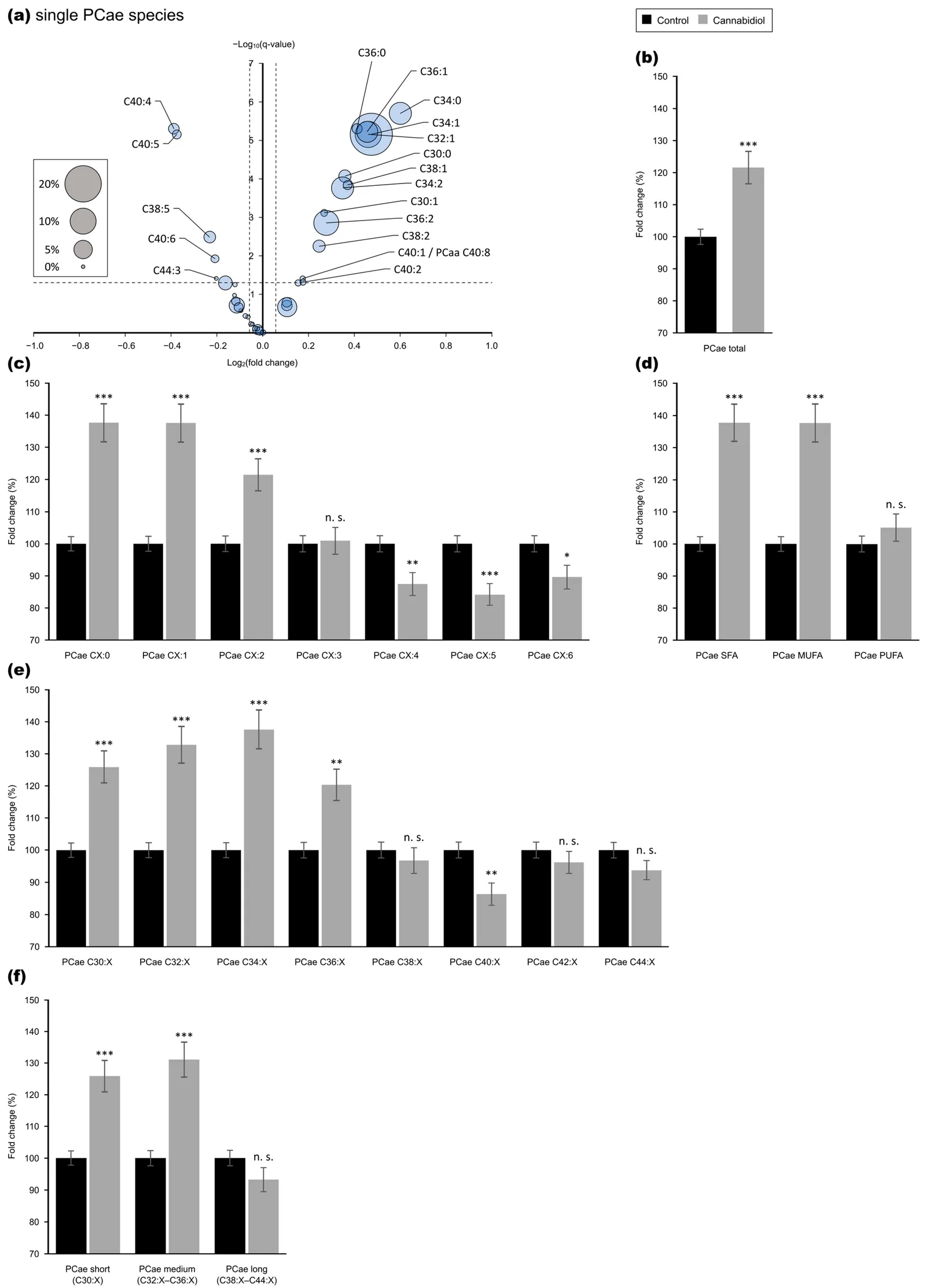
PCae-category signals in homogenates of SH-SY5Y wt cells incubated with 1 µM CBD for 72 h compared to the solvent control. (**a**) Fold changes of the individual PCae species are shown as a volcano plot. The bubble area represents the proportion of each species in the PCae lipid class in the CBD-treated group. Gray bubbles show reference proportions. The vertical dashed lines represent the mean SEM, and the horizontal line marks q = 0.05. Significantly changed species are named. (**b**–**f**) Bar charts showing the relative fold change of the PCae total (**b**), the relative fold changes of PCae species grouped by their saturation (**c**) or grouped by classes of saturation (**d**) and the relative fold changes of PCae species grouped by their chain length (**e**) or grouped by classes of chain length (**f**). Error bars represent the SEM. Significance classifications are based on BH-adjusted *p*-values (q): * q ≤ 0.05, ** q ≤ 0.01, and *** q ≤ 0.001; n. s. = non-significant. For the class total in panel (**b**), the same thresholds refer to the Welch’s *t*-test *p*-value. The statistical analyses applied are described in detail in Section 2.5.3. The corresponding Welch’s *t*-test and BH-adjusted *p*-values are provided in Supplementary Table S7. The underlying n-counts are listed in Supplementary Table S8.

**Figure 3:**
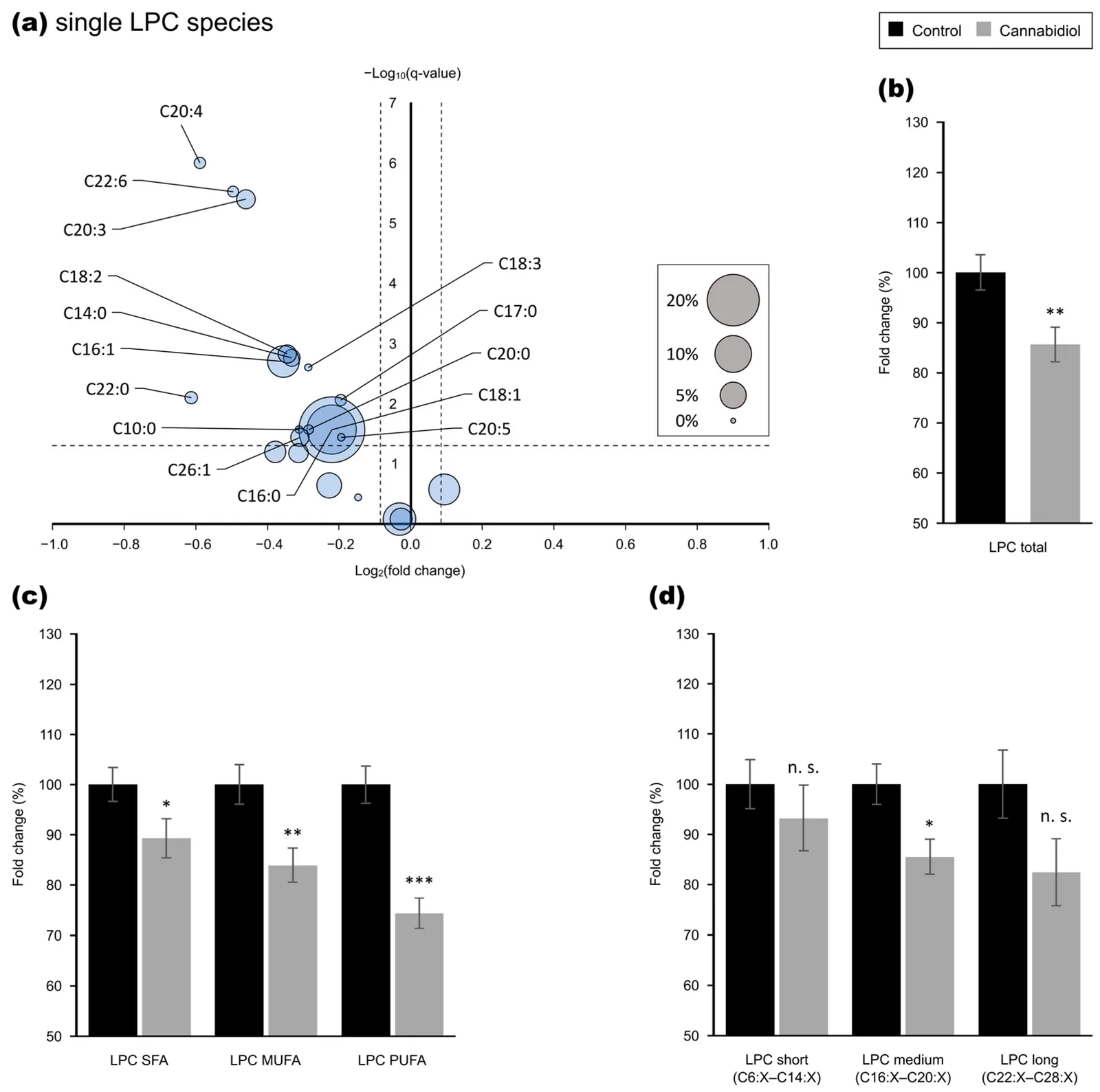
LPC levels in homogenates of SH-SY5Y wt cells incubated with 1 µM CBD for 72 h compared to the solvent control. (**a**) Fold changes of the individual LPC species are shown as a volcano plot. The bubble area represents the proportion of each species in the LPC lipid class in the CBD-treated group. Gray bubbles show reference proportions. The vertical dashed lines represent the mean SEM, and the horizontal line marks q = 0.05. Significantly changed species are named. (**b**–**d**) Bar charts showing the relative fold change of the LPC total (**b**), the relative fold changes of LPC species grouped by classes of saturation (**c**) and the relative fold changes of LPC species grouped by classes of chain length (**d**). Error bars represent the SEM. Significance classifications are based on BH-adjusted *p*-values (q): * q ≤ 0.05, ** q ≤ 0.01, and *** q ≤ 0.001; n. s. = non-significant. For the class total in panel (**b**), the same thresholds refer to the Welch’s *t*-test *p*-value. The statistical analyses applied are described in detail in Section 2.5.3. The corresponding Welch’s *t*-test and BH-adjusted *p*-values are provided in Supplementary Table S7. The underlying n-counts are listed in Supplementary Table S8.

**Figure 4:**
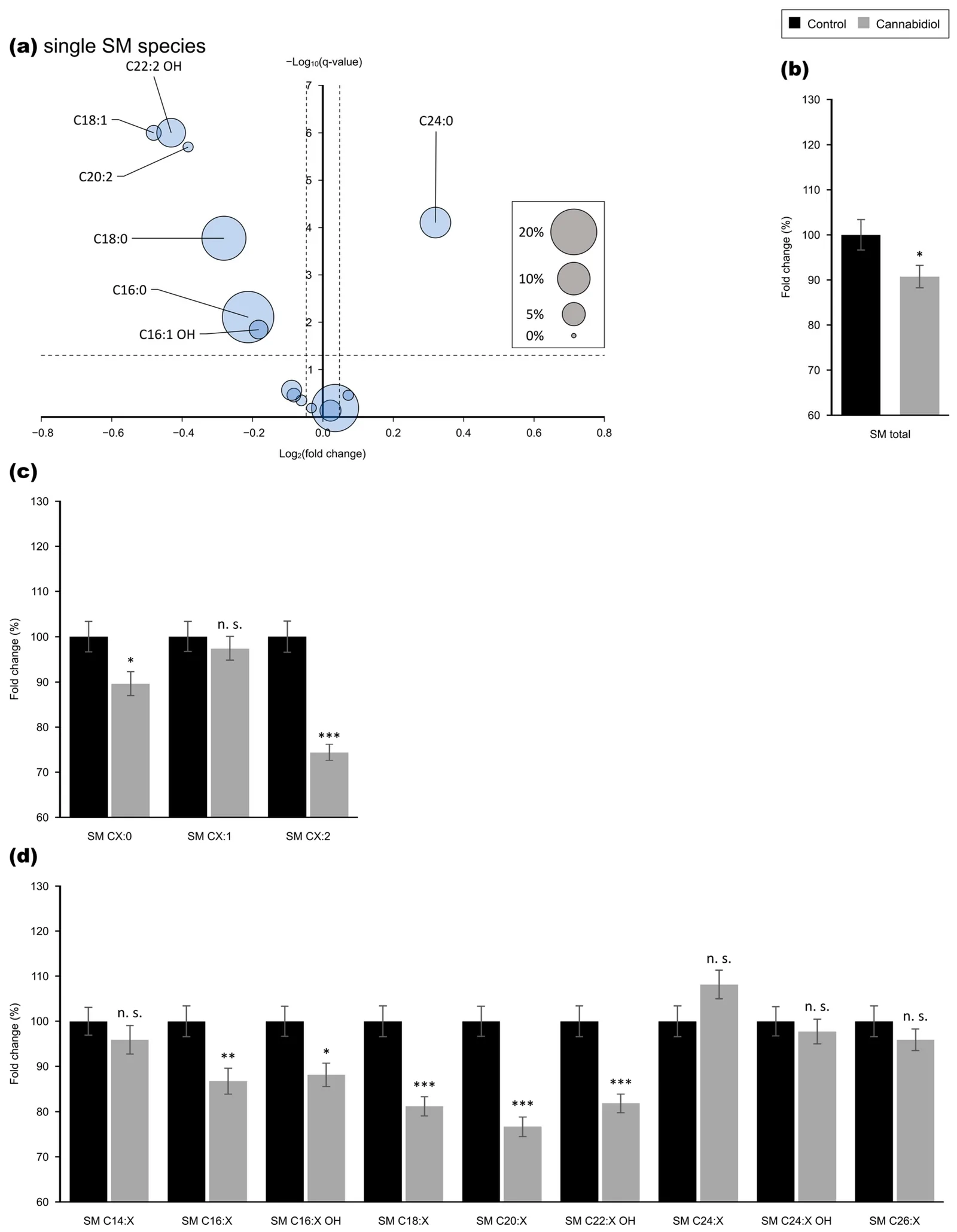
SM levels in homogenates of SH-SY5Y wt cells incubated with 1 µM CBD for 72 h compared to the solvent control. (**a**) Fold changes of the individual SM species are shown as a volcano plot. The bubble area represents the proportion of each species in the SM lipid class in the CBD-treated group. Gray bubbles show reference proportions. The vertical dashed lines represent the mean SEM, and the horizontal line marks q = 0.05. Significantly changed species are named. (**b**–**d**) Bar charts showing the relative fold change of the SM total (**b**), the relative fold changes of SM species grouped by their saturation (**c**) and the relative fold changes of SM species grouped by their chain length (**d**). Error bars represent the SEM. Significance classifications are based on BH-adjusted *p*-values (q): * q ≤ 0.05, ** q ≤ 0.01, and *** q ≤ 0.001; n. s. = non-significant. For the class total in panel (**b**), the same thresholds refer to the Welch’s *t*-test *p*-value. The statistical analyses applied are described in detail in Section 2.5.3. The corresponding Welch’s *t*-test and BH-adjusted *p*-values are provided in Supplementary Table S7. The underlying n-counts are listed in Supplementary Table S8.

**Figure 5:**
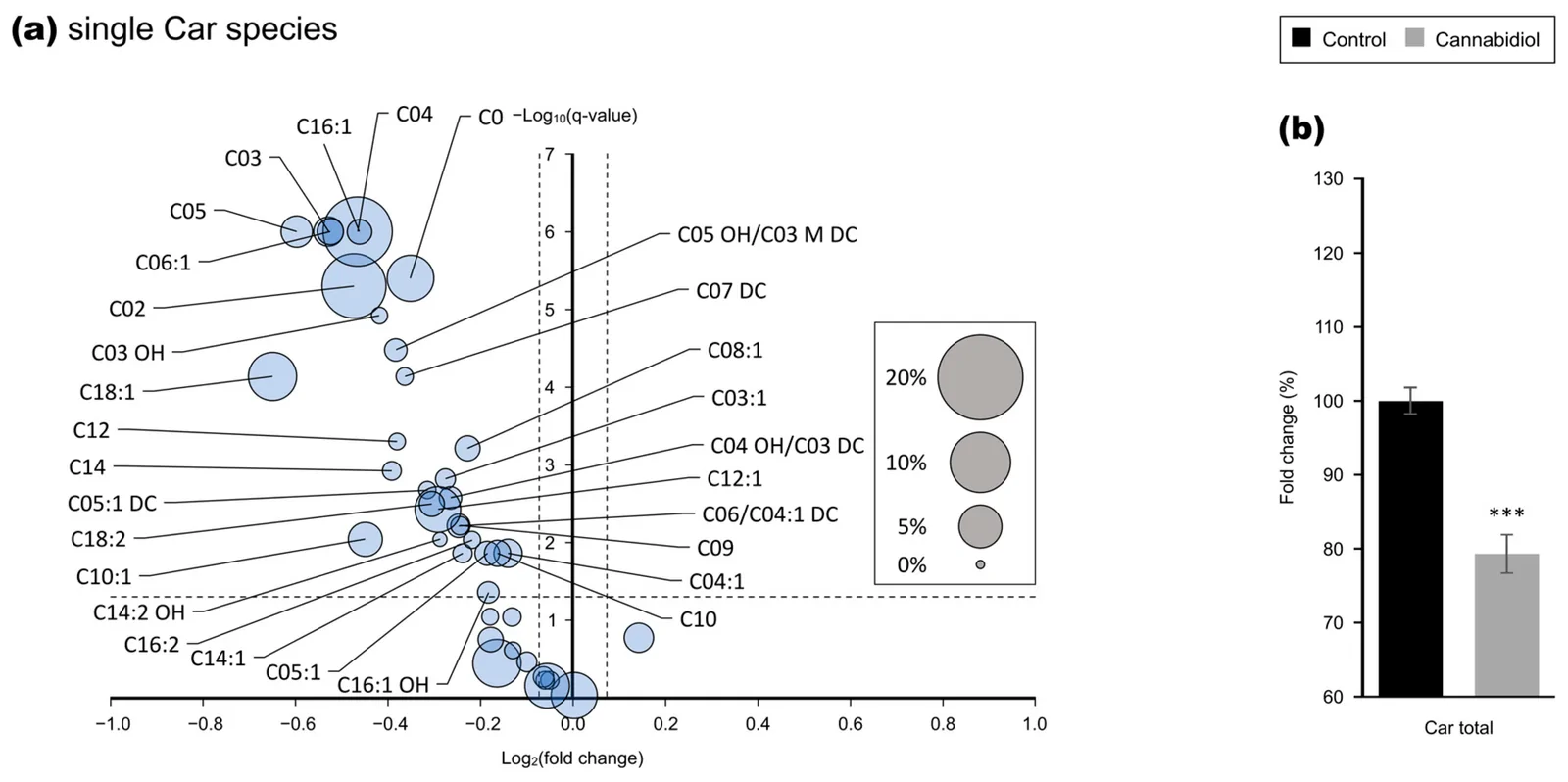
Carnitine-related analytes in homogenates of SH-SY5Y wt cells incubated with 1 µM CBD for 72 h compared to the solvent control. (**a**) Fold changes of the individual Car species are shown as a volcano plot. The bubble area represents the proportion of each species in the Car lipid class in the CBD-treated group. Gray bubbles show reference proportions. The vertical dashed represents the mean SEM, and the horizontal line marks q = 0.05. Significantly changed species are named. (**b**) Bar chart showing the relative fold change of the Car total. Error bars represent the SEM. Individual-analyte significance in panel (**a**) is based on BH-adjusted *p*-values (q ≤ 0.05). The class total in panel (**b**) is evaluated using a Welch’s *t*-test *p*-value (*** *p* ≤ 0.001). The statistical analyses applied are described in detail in Section 2.5.3. The corresponding Welch’s *t*-test and BH-adjusted *p*-values are provided in Supplementary Table S7. The underlying n-counts are listed in Supplementary Table S8.

To analyze measurements from different culture runs, samples were normalized on the primarily measured control group by comparison of the arithmetic mean of each control group. The arithmetic mean was calculated for each lipid species within each CBD or solvent-control condition, and the mean of the corresponding solvent-control group was defined as 100%. For the 1 µM/72 h reference condition, control and CBD groups were compared for each lipid species using unpaired Welch’s *t*-tests, followed by Benjamini–Hochberg (BH) adjustment of the resulting *p*-values to control the false discovery rate (FDR) at 5% within each lipid class. The comparison families followed the biochemically defined lipid classes analyzed separately in Figures 1–5. Grouped endpoints were adjusted separately within each saturation or chain-length set. The class-total comparison in each figure is reported with its Welch’s *t*-test *p*-value.

These results are shown in bar charts. Fold changes of the lipid species within a lipid class were plotted on a binary-logarithmic abscissa in the main volcano plots, with BH-adjusted *p*-values (q) plotted as the negative common logarithm on the ordinate. For display, q values below 0.000001 are plotted at 0.000001. Bubble area represents the proportion of each lipid species within its lipid class under CBD exposure; a minimum bubble size was used for visualization, as indicated by the gray reference bubbles. The vertical dashed lines represent the mean standard error of the mean (mean SEM) as a descriptive guide and do not define statistical significance. For individual species and grouped endpoints in the main figures, significance was defined as * q ≤ 0.05, ** q ≤ 0.01, and *** q ≤ 0.001. Here, *p* denotes the Welch’s *t*-test *p*-value before multiple-testing adjustment, and q denotes the BH-adjusted *p*-value. The complementary PCaa and PCae datasets and Supplementary Figures S3–S6 retained nominal significance classifications based on Welch’s *t*-test *p*-values for comparison. Supplementary Table S7 presents Welch’s *t*-test and BH-adjusted *p*-values for the 1 µM/72 h reference condition and Welch’s *t*-test *p*-values for the additional PCaa and PCae conditions.

## 3. Results

### 3.1. Study Design

Undifferentiated SH-SY5Y wt cells were exposed to 1 µM CBD or solvent control for 72 h to characterize CBD-associated changes in the measured cellular lipid profile. Cell homogenates were adjusted to 8 mg/mL protein before solid–liquid extraction and targeted semiquantitative mass-spectrometric analysis. Signals were normalized to the corresponding internal standards and expressed relative to the solvent-control group. The targeted panel comprised 159 lipid and carnitine-related analytes: 43 PCaa, 39 PCae, 22 LPC, 14 sphingomyelins, and 41 carnitine-related analytes, including free carnitine and acylcarnitines. The distribution of the measured analytes is summarized in Supplementary Table S6.

### 3.2. Diacyl-Phosphatidylcholines

Diacyl phosphatidylcholine sum species (PCaa) are major phospholipid components of eukaryotic cellular membranes. PCaa species are assigned as containing two ester-linked chains; in the present annotation, total carbon number and degree of unsaturation are reported without assignment of individual fatty-acyl chains or sn-positions.

After incubation with 1 µM CBD, 27 out of the 43 measured PCaa species showed higher mean abundances, and eight of these changes met the BH-adjusted significance criterion (Section 2.5.3). Sixteen species showed lower mean abundances, and four of these changes met the same criterion. The species that increased included the PCaa signals with the largest relative abundances, which together represented more than 95% of the summed relative PCaa abundance (Figure 1a). The eight significantly increasing species accounted for approximately 68% of the summed relative PCaa abundance in the CBD-treated cells (C32:1, C34:1, C36:0, C36:1, C36:2, C38:1, C38:3, and C40:2). Accordingly, the summed relative PCaa abundance increased to 121.9% of the solvent-control value (±5.8%, *p* = 0.003; Figure 1b). Analysis according to total degree of unsaturation showed increases in monounsaturated (129.9% ± 6.3%, q = 0.0017) and diunsaturated species (119.2% ± 5.6%, q = 0.0202; Figure 1c). Saturated PCaa and the combined polyunsaturated PCaa category showed smaller changes (saturated: 111.0% ± 5.6%, q = 0.0945; polyunsaturated: 115.5% ± 5.4%, q = 0.0291; Figure 1d). An increase was particularly evident for species with intermediate total carbon numbers C32:X–C36:X (123.6% ± 5.9%, q = 0.0047; Figure 1f), with significant increases in C34:X (122.3% ± 5.9%, q = 0.0176) and C36:X (135.1% ± 6.4%, q ≤ 0.001; Figure 1e). In summary, the most abundant PCaa species and several species with intermediate total carbon numbers increased, whereas a subset of lower-abundance species declined.

### 3.3. Ether-Containing Phosphatidylcholine Sum Species

PCae represents the operational analytical category used for nominally ether-containing phosphatidylcholine sum species in the present targeted assay. The exact ether-bond type and individual fatty-acyl composition were not structurally resolved, and isobaric highly unsaturated diacyl phosphatidylcholine compositions cannot be excluded for specified signals. The results are therefore interpreted according to the total carbon number and total degree of unsaturation rather than as structurally confirmed plasmalogens.

Under the influence of 1 µM CBD, 18 out of 39 PCae-category signals increased, 13 of which met the BH-adjusted significance criterion (Section 2.5.3). Twenty-one signals decreased, and five of these changes met the same criterion. All signals contributing individually more than 4% of the summed relative PCae abundance increased (Figure 2a), and the summed relative abundance increased to 121.6% of the solvent-control value (±5.0%, *p* ≤ 0.001; Figure 2b). According to total degree of unsaturation, signals categorized as saturated increased to 137.7% (±5.9%, q ≤ 0.001) and monounsaturated signals to 137.6% (±5.9%, q ≤ 0.001; Figure 2c). Diunsaturated signals increased to 121.5% (±5.0%, q ≤ 0.001), whereas signals with three double-bond equivalents had a mean abundance of 101.0% of the control (±4.2%, q = 0.8406). Signals with four or more double-bond equivalents declined, most strongly for CX:5 (84.2% ± 3.4%, q ≤ 0.001) and CX:6 (89.7% ± 3.7%, q = 0.0296; Figure 2c). Because of the proportional predominance of the diunsaturated signals, the combined polyunsaturated category had a mean abundance of 105.1% of the control (±4.2%, q = 0.3001; Figure 2d). According to total carbon number, increases were observed for C30:X (125.9% ± 5.0%, q ≤ 0.001), C32:X (132.9% ± 5.7%, q ≤ 0.001), C34:X (137.6% ± 6.0%, q ≤ 0.001), and C36:X (120.3% ± 4.9%, q = 0.0012; Figure 2e,f). C38:X (96.8% ± 4.0%, q = 0.4955), C42:X (96.2% ± 3.4%, q = 0.4209), and C44:X (93.7% ± 3.0%, q = 0.1388) remained close to the solvent-control values, whereas C40:X decreased to 86.3% (±3.5%, q = 0.0035; Figure 2e), reflecting lower C40:4 (76.5% ± 3.1%, q ≤ 0.001) and C40:5 signals (77.1% ± 3.4%, q ≤ 0.001; Figure 2a). Taken together, the most abundant PCae signals and several species with lower total carbon numbers and lower degrees of unsaturation increased, whereas several signals with higher total carbon numbers and higher degrees of unsaturation declined.

### 3.4. Lyso-Phosphatidylcholines

Lysophosphatidylcholines contain one fatty-acyl chain and participate in phosphatidylcholine remodeling and cellular signaling. Some LPC species can serve as transport forms for polyunsaturated fatty acids. Because double-bond positions were not resolved by the present method, LPC C22:6 is compatible with, but cannot be identified unambiguously as a DHA-containing LPC species.

Twenty-one out of the 22 detected LPC species were reduced after treatment with 1 µM CBD, 15 of which were significantly reduced. C20:4 (66.5% ± 3.0%, q ≤ 0.001), C22:6 (70.9% ± 3.1%, q ≤ 0.001), and C20:3 (72.7% ± 3.1%, q ≤ 0.001) showed pronounced reductions (Figure 3a). For the LPC class as a whole, there was a significant decrease to 85.7% (±3.5%, *p* = 0.005, Figure 3b). Saturated species showed a smaller decrease to 89.3% (±3.9%, q = 0.0427) than monounsaturated or polyunsaturated species with 83.9% (±3.4%, q = 0.0044) and 74.4% (±3.0%, q ≤ 0.001, Figure 3c).

LPC with short chain lengths (C6:X–C14:X) decreased non-significantly to 93.2% (±6.6%, q = 0.4102), medium chain lengths (C16:X–C20:X) decreased significantly to 85.5% (±3.5%, q = 0.0260), and long chain lengths (C22:X–C28:X) had a mean abundance of 82.4% of the control (±6.7%, q = 0.1060, Figure 3d). With one exception, all LPC species showed lower mean abundances, with larger mean reductions in the more unsaturated groups.

### 3.5. Sphingomyelins

Sphingomyelins (SM) are major structural components of cellular membranes and are preferentially localized within lipid rafts, where they regulate membrane order and signal transduction. In the nervous system, SM are abundant in neuronal membranes and myelin sheaths, and alterations in their composition have been linked to synaptic dysfunction and neurodegeneration.

Ten out of fourteen SM species showed a reduction after incubation with 1 µM CBD, six decreased significantly (C16:0, C16:1 OH, C18:0, C18:1, C20:2, C22:2 OH). Four species increased, with C24:0 being significantly elevated (Figure 4a). Overall, SM decreased significantly to 90.7% (±2.5%, *p* = 0.031, Figure 4b).

Saturated species decreased only slightly to 89.6% (±2.6%, q = 0.0267), monounsaturated species had a mean abundance of 97.3% of the control (±2.6%, q = 0.5315), while double unsaturated species decreased substantially to 74.4% (±1.8%, q ≤ 0.001, Figure 4c).

Relative fold changes also varied across chain-length groups. Chain-length groups C14:X, C24:X, C24:X OH and C26:X showed mean values close to the control, while C16:X–C22:X OH decreased significantly, with a particularly strong effect in the case of C18:X (81.2% ± 2.1%, q ≤ 0.001), C20:X (76.7% ± 2.2%, q ≤ 0.001), and C22:X OH (81.8% ± 2.1%, q ≤ 0.001, Figure 4d).

### 3.6. Carnitine and Acylcarnitines

Free carnitine and acylcarnitines participate in cellular acyl-group transport and metabolism. Their steady-state abundances are influenced by acyl-CoA availability, carnitine acyltransferase reactions, mitochondrial transport, subsequent metabolic utilization, and cellular turnover. Consequently, cellular carnitine and acylcarnitine pool sizes alone do not quantify the direction or rate of mitochondrial fatty-acid oxidation.

Following CBD exposure, 41 carnitine-related analytes were measured. Thirty-nine showed lower mean abundances, and 29 of these changes met the BH-adjusted significance criterion (Section 2.5.3). Two analytes showed higher mean abundances. The summed relative abundance of the carnitine-related analytes was reduced to 79.4% (±2.6%, *p* ≤ 0.001) of the solvent-control value (Figure 5).

### 3.7. Evaluation of the Phosphatidylcholine Response Under Additional Experimental Conditions

To evaluate the central phosphatidylcholine response under additional exposure conditions, PCaa and PCae were analyzed in SH-SY5Y wt cells exposed to 0.25 µM CBD for 24 or 72 h and to 1 µM CBD for 24 h. The comparison incorporated the 1 µM/72 h dataset as the reference condition.

After 24 h of exposure to 1 µM CBD, six PCaa and eleven PCae species met the nominal significance criterion (Welch’s *t*-test *p* ≤ 0.05; Section 2.5.3). All 17 species changed in the same direction after 72 h. Of the species meeting this nominal criterion after 72 h, 12 out of 16 PCaa and 19 out of 21 PCae species already showed concordant directional changes after 24 h.

Directional concordance across concentrations was also evident. At 0.25 µM CBD after 24 h, 35 out of 43 PCaa and 34 out of 39 PCae species changed in the same direction as under the corresponding 1 µM exposure. Among these directionally concordant species, the deviation from the respective solvent control was greater at 1 µM for 29 PCaa and 32 PCae species. After 72 h, 30 out of 43 PCaa and 28 out of 39 PCae species showed concordant directions at 0.25 and 1 µM CBD; among these, the deviation was greater at 1 µM for 23 out of 30 PCaa and 27 out of 28 PCae species. At 1 µM CBD, the phosphatidylcholine response was already evident after 24 h, and the 72 h reference condition involved a broader set of species. At 0.25 µM CBD, the changes were smaller but predominantly directionally concordant with those observed under the corresponding 1 µM conditions. The complete comparison is presented in Supplementary Figures S3–S6 and Supplementary Table S7.

The central phosphatidylcholine response was additionally examined in stably APPswe-expressing SH-SY5Y cells exposed to 1 µM CBD for 72 h. Eighteen PCaa and sixteen PCae species met the nominal significance criterion (Welch’s *t*-test *p* ≤ 0.05; Section 2.5.3), and all of these species showed higher relative abundances following CBD exposure. These findings show that both phosphatidylcholine classes also responded in an APPswe-expressing cellular context. The partly different descriptive species-level response pattern is compatible with a response shaped by the cellular background; no formal genotype-by-treatment interaction was tested. The results are not interpreted as evidence of altered APP processing, Aβ production, or disease modification. The complete results are presented in Supplementary Figures S3–S6 and Supplementary Table S7.

## 4. Discussion

Exposure of undifferentiated SH-SY5Y wt cells to CBD produced broad, class-specific remodeling across the measured cellular lipid panel. Summed relative abundances of PCaa and PCae-category signals increased, whereas LPC, sphingomyelins, free carnitine, and most measured acylcarnitines declined. Within the phosphatidylcholine classes, many species with lower total degrees of unsaturation increased, while several highly unsaturated PCae-category signals and polyunsaturated LPC species decreased. This combined species-level pattern is consistent with a relative shift toward a less polyunsaturated profile within the measured lipid panel in wt cells.

The additional datasets reinforced the central phosphatidylcholine finding across further experimental conditions. At 1 µM CBD, PCaa and PCae changes were already apparent after 24 h; all 17 species meeting the nominal Welch’s *t*-test criterion (*p* ≤ 0.05) at this time point changed in the same direction after 72 h, and the 72 h reference condition involved a broader set of species. At 0.25 µM CBD, the changes were smaller but predominantly directionally concordant with those observed under the corresponding 1 µM conditions. In stably APPswe-expressing cells, 18 PCaa and 16 PCae species met the nominal Welch’s *t*-test criterion (*p* ≤ 0.05), all with higher relative abundances following CBD exposure, demonstrating that both phosphatidylcholine classes also responded in an amyloidogenic genetic context. The partly different descriptive species-level pattern is compatible with a response shaped by the cellular background; no formal genotype-by-treatment interaction was tested.

CBD-associated changes in phospholipid and sphingolipid metabolism have previously been reported in other experimental systems [49,50,51]. The present study extends these observations to an undifferentiated human neuroblastoma-derived cell system and identifies, within the same targeted analysis, a broad response spanning phosphatidylcholines, lysophosphatidylcholines, sphingomyelins, and carnitine-related analytes. The combined profile therefore indicates that CBD-associated lipid remodeling is not confined to an individual lipid species or class.

The increased abundances of several phosphatidylcholine sum species with lower degrees of unsaturation may reflect altered regulation of lipid synthesis, desaturation, remodeling, uptake, or turnover. PPARγ-associated regulation of SCD1 can influence fatty-acid desaturation [52,53], while CBD has been associated with PPARγ-dependent signaling in other experimental systems [21,22,23]. These observations provide a biologically plausible framework in which PPARγ- and SCD1-related pathways may contribute to the observed phosphatidylcholine response. Future work integrating PPARγ and SCD1 expression analyses with measurements of PPARγ transcriptional activity and SCD1 desaturase activity would provide a direct test of this mechanistic framework.

The summed relative abundance of the PCae analytical category increased overall, although the response differed with total degree of unsaturation: signals with lower degrees of unsaturation generally increased, whereas several highly unsaturated signals declined. The analytical method did not distinguish plasmanyl from plasmenyl phosphatidylcholines, and isobaric highly unsaturated PCaa compositions cannot be excluded for specified transitions. Accordingly, these analytes are interpreted as PCae-category sum-composition signals rather than structurally confirmed plasmalogens, and individual DHA- or arachidonic-acid-containing phosphatidylcholines cannot be assigned. Structurally resolved lipidomic analyses will be valuable for defining the ether-bond and fatty-acyl compositions underlying this prominent response.

The observed redistribution among lipid species with different degrees of unsaturation may have consequences for cellular responses to oxidative membrane stress. Highly polyunsaturated phospholipids are susceptible to lipid peroxidation, and specific PUFA-containing phospholipids contribute to ferroptotic sensitivity [54,55]. Membrane lipid unsaturation can also influence cellular stress responses [56]. The observed compositional redistribution therefore provides a rationale to test whether CBD-associated lipid remodeling alters cellular susceptibility to lipid peroxidation. The present lipidomic profile alone does not establish a ferroptosis-resistant or oxidative-stress-resistant state; direct lipid-peroxidation measurements combined with ferroptosis-induction and pharmacological-rescue experiments are required to determine whether this compositional response modifies ferroptotic sensitivity.

The reduction in intracellular LPC C22:6 is of potential biological interest because this sum composition is compatible with a DHA-containing LPC, although its double-bond positions were not resolved. Circulating LPC-bound DHA is an established substrate for MFSD2A-mediated transport across the blood–brain barrier [57,58,59], placing the observed intracellular change in a biologically relevant context of DHA-related LPC metabolism. However, intracellular LPC C22:6 abundance does not determine extracellular LPC-DHA availability or transport across the blood–brain barrier. This potential connection can be examined through measurements of extracellular LPC species and experiments in appropriate blood–brain barrier models.

Lower mean abundances for 39 out of the 41 measured carnitine-related analytes, including free carnitine, indicate a pronounced change in the cellular carnitine/acylcarnitine pool. These metabolites integrate several interconnected processes, including acyl-CoA availability, acylcarnitine formation, mitochondrial transport, subsequent utilization, and cellular turnover. The steady-state profile therefore identifies altered carnitine/acylcarnitine homeostasis and cellular acyl-group handling as clear targets for functional investigation. Reports of CBD-associated changes in mitochondrial respiration in other experimental systems [60,61,62] provide a rationale for follow-up integrating stable-isotope tracing [63], targeted substrate-supported respirometry, and expression analyses of carnitine palmitoyltransferase 1 (CPT1) and β-hydroxyacyl-CoA dehydrogenase (β-HAD). Together, these approaches can define the relationship between the observed steady-state pool and mitochondrial fatty-acid oxidation flux.

The broad reduction in sphingomyelin abundance adds a sphingolipid component to the CBD-associated cellular lipid response. Sphingomyelins contribute to cellular membranes and lipid-raft organization and are also abundant in myelin. Moreover, experimental inhibition of sphingomyelin synthase 1 has been reported to promote lysosomal degradation of BACE1 and reduce amyloid pathology in APP/PS1 models [64]. The present findings therefore provide a rationale for testing whether CBD-associated sphingolipid remodeling intersects with amyloidogenic processing or myelin-related biology. The current whole-cell measurements do not themselves demonstrate effects on myelin, sphingomyelin synthase activity, BACE1, APP processing, or Aβ production; these questions require dedicated amyloid-processing and myelination models.

Several of the affected lipid classes have repeatedly been implicated in AD-related lipid dysregulation [30,31,32,65,66,67,68,69,70,71,72]. Together with the observation of phosphatidylcholine remodeling in APPswe-expressing cells, these associations place the CBD response within an AD-relevant lipid and amyloidogenic context. The results thereby define specific hypotheses concerning oxidative membrane susceptibility, cellular acyl-group handling, sphingolipid-related amyloidogenic pathways, and the context dependence of the phosphatidylcholine response. These associations support the functional testing of AD-related consequences, but do not establish neuroprotection, normalization of an AD phenotype, or disease modification.

The interpretive scope of the study is defined by the cellular systems and the targeted analytical design. Undifferentiated SH-SY5Y wt cells provide a controlled human neuroblastoma-derived system for examining cell-autonomous lipid responses but do not reproduce mature primary neurons or the multicellular environment of the human brain. APPswe expression adds an AD-relevant amyloidogenic background but does not constitute a complete model of the complex and progressive pathology of AD. The targeted analytical design captured complementary aspects of phosphatidylcholine remodeling, sphingomyelin composition, and cellular acyl-group handling; the findings therefore characterize CBD-associated remodeling within these measured lipid groups.

Whole-cell homogenates integrate lipid abundances across cellular compartments and consequently do not establish membrane-specific or subcellular localization. Likewise, the sum-composition and steady-state measurements do not resolve individual fatty-acyl structures or directly determine pathway activity, enzyme activity, metabolic flux, mitochondrial function, or AD-related protein endpoints such as APP processing and tau phosphorylation. Although CBD-treated and solvent-control cells were maintained under identical serum-reduced conditions, the contributions of serum-derived lipids and endogenous synthesis, remodeling, degradation, uptake, and release cannot be separated fully. The brief terminal HPLC-grade water washes were performed identically in all groups and are consistent with related adherent-cell mass-spectrometric workflows [39,40]. Nevertheless, because water is hypotonic, subtle or transient osmotic effects and an influence on absolute analyte recovery cannot be excluded despite the absence of overt morphological changes during the washing procedure.

The additional experiments evaluated the two central phosphatidylcholine classes at a lower CBD concentration, after a shorter exposure period, and in APPswe-expressing cells. Because the selected conditions did not constitute a formal factorial experimental design, concentration-by-time and genotype-by-treatment interactions cannot be derived, with the 1 µM/72 h dataset serving as the reference condition. Within these defined boundaries, the breadth of the 1 µM/72 h lipid response and the directional coherence of the additional phosphatidylcholine data strengthen the conclusion that CBD exposure remodels the measured cellular lipid profile and provide a focused basis for mechanistic investigation in more complex neurodegeneration-relevant models. Future studies integrating the lipidomic readouts with analyses of PPARγ/SCD1 signaling, fatty-acid oxidation, and total and phosphorylated tau could determine the molecular and functional consequences of the observed lipid remodeling.

## 5. Conclusions

CBD induced broad, class-specific remodeling of the measured lipid profile in SH-SY5Y wt cells. Summed relative PCaa and PCae abundances increased, whereas LPC, sphingomyelins, free carnitine, and most measured acylcarnitines declined. At the species level, the combined pattern in wt cells was consistent with a relative shift toward a less polyunsaturated profile within the measured lipid panel. Additional experiments showed that the principal phosphatidylcholine response was already apparent after 24 h, was smaller but directionally coherent at the lower CBD concentration, and was also observed in stably APPswe-expressing SH-SY5Y cells. Because the affected lipid classes have been implicated in AD-related lipid dysregulation, these findings provide an AD-relevant lipid context for further investigation. Functional studies in differentiated, primary, multicellular, and in vivo models will be required to determine the underlying mechanisms and biological consequences of this response.

## Data Availability

The original contributions presented in this study are included in the article/Supplementary Materials. Further inquiries can be directed to the corresponding author.

## Supplementary Materials

The following supporting information can be downloaded at: https://www.mdpi.com/article/10.3390/cells15181714/s1, Figure S1: Plasma-membrane integrity during the preliminary CBD concentration screen, assessed by LDH release; Figure S2: Total cellular protein content in SH-SY5Y wt samples after exposure to 1 µM CBD or solvent control for 72 h before adjustment to a protein concentration of 8 mg/mL; Figure S3: PCaa abundance patterns across CBD concentrations and incubation periods in wt and APPswe-expressing SH-SY5Y cells; Figure S4: PCaa volcano plots for SH-SY5Y wt and APPswe-expressing cells versus matched solvent controls; Figure S5: PCae abundance patterns across CBD concentrations and incubation periods in wt and APPswe-expressing SH-SY5Y cells; Figure S6: PCae volcano plots for SH-SY5Y wt and APPswe-expressing cells versus matched solvent controls; Table S1: Internal standards added before lipid extraction and used for normalization; Table S2: Measurement parameters of the 4000-quadrupole linear-ion trap (Qtrap); Table S3: Measurement parameters of the Orbitrap ID-X; Table S4: MRM pairs of the detected lipid species in the 4000-quadrupole linear-ion trap (Qtrap); Table S5: Monitored precursor/product-ion pairs of the detected lipid species in the Orbitrap ID-X; Table S6: Overview of the lipid and carnitine-related analyte groups covered by the targeted analytical panel; Table S7: Welch’s *t*-test *p*-values and Benjamini–Hochberg (BH)-adjusted *p*-values (q) for the wt 1 µM/72 h reference dataset, and Welch’s *t*-test *p*-values for the additional PCaa and PCae conditions; Table S8: Number of biological replicates and culture runs for all reported lipidomic experiments; Table S9: Complete results for the 1 µM/72 h dataset without lipid-class-specific sample exclusion.
