# Supplementary material for: Lipidomic Evidence for Cannabidiol-Induced Remodeling of Cellular Lipid Composition in SH-SY5Y Cells
Source: Cells. 2026 Sep 21;15(18):1714. doi: 10.3390/cells15181714 (PMC13605370; doi:10.3390/cells15181714)
Supplement: Supplementary file 1 [file cells-15-01714-s001.zip › cells-4433234-supplementary.pdf]

Supplementary material

Lipidomic Evidence for Cannabidiol-Induced Remodeling of Cellular Lipid Composition in SH-SY5Y Cells

Oliver Walzer [1,†], Vincent Konrad Johannes Erhardt [1,†], Jill Sven René Zügner [1], Natalya V. Izarova [1], Kristina Endres [2,3], Heike Sabine Grimm [1,4], Tobias Hartmann [1,5] and Marcus Otto Walter Grimm [1,4,*]

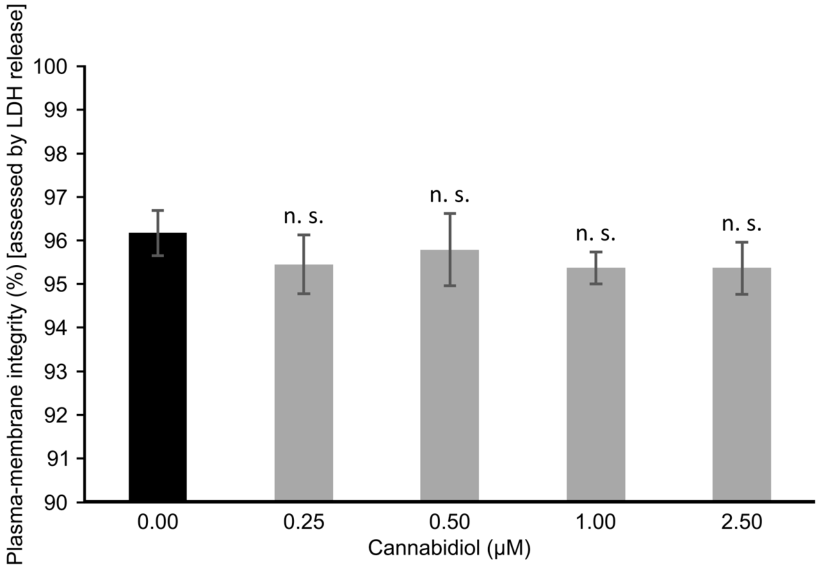

Figure S1. Plasma-membrane integrity during the preliminary CBD concentration screen, assessed by LDH release. SH-SY5Y wt cells were incubated with the indicated CBD concentrations for 72 h or with solvent control (0.00 µM). The plotted values were derived from LDH release into the culture medium. Error bars represent the standard error of the mean; n.s. indicates no statistically significant difference from solvent control, calculated by unpaired two sample Welch t-test. The numbers of individual biological samples are from left to right 4, 4, 4, 3, 3.

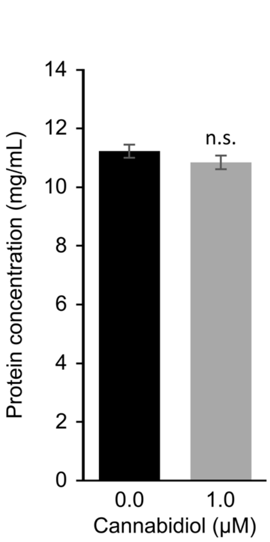

Figure S2. Total cellular protein content in SH-SY5Y wt samples after exposure to 1 µM CBD or solvent control for 72 h before adjustment to a protein concentration of 8mg/ml.
Protein content was determined by BCA assay as described in section 2.4, samples were measured in technical triplicates and averaged to one data point for every biological n, which was represented by one cell culture dish. n is 65 for control and 34 for CBD. Error bars represent the standard error of the mean; n.s. indicates no statistically significant difference from the corresponding solvent control, calculated by unpaired two sample Welch t-test.

Species	SH-SY5Y-wt 0.25µM 24h	SH-SY5Y-wt 0.25µM 72h	SH-SY5Y-wt 1µM 24h	SH-SY5Y-wt 1µM 72h	SH-SY5Y-APPswe 1µM 72h
PC aa C20:0	102.4	97.8	103.6	104.1	95.7
PC aa C24:0	99.9	103.6	102.5	98.8	120.9
PC aa C26:0	105.8	95.6	102.8	96.6	124.6
PC aa C28:0	108.3	101.4	110.9	111.7	116.1
PC aa C28:1	106.7	100.7	107.5	103.4	113.4
PC aa C30:0	106.1	102.6	109.7	110.6	111.9
PC aa C30:2	104.9	100.0	102.0	90.1	108.1
PC aa C32:0	105.6	102.1	106.1	106.8	100.3
PC aa C32:1	106.7	106.2	108.7	120.4	117.6
PC aa C32:2	104.5	105.6	98.7	99.7	112.2
PC aa C32:3	104.1	97.4	89.3	77.4	109.0
PC aa C34:1	103.9	106.1	109.1	126.2	114.3
PC aa C34:2	103.6	105.7	101.1	115.5	112.9
PC aa C34:3	103.9	103.8	92.9	95.9	106.5
PC aa C34:4	109.1	101.1	98.9	95.2	110.8
PC aa C36:0	107.8	109.4	120.1	162.1	134.0
PC aa C36:1	108.0	111.0	121.3	159.8	135.0
PC aa C36:2	103.3	106.9	105.7	126.8	114.8
PC aa C36:3	106.9	107.6	102.2	111.3	114.7
PC aa C36:4	112.0	105.5	114.8	102.5	114.0
PC aa C36:5	108.9	104.1	109.6	95.0	110.2
PC aa C36:6	112.6	106.1	112.8	106.8	113.6
PC aa C38:0	102.9	97.9	99.9	87.7	94.6
PC aa C38:1	104.8	105.2	108.1	136.2	114.0
PC aa C38:3	105.5	108.1	108.4	123.7	119.5
PC aa C38:4	107.7	103.4	113.9	109.1	113.9
PC aa C38:5	109.7	102.9	114.4	102.0	108.7
PC aa C38:6	110.4	104.4	114.4	103.4	108.8
PC aa C40:0	105.2	96.5	108.2	84.6	105.3
PC aa C40:1	104.2	101.3	102.4	97.2	100.6
PC aa C40:2	106.8	102.7	106.7	124.8	115.9
PC aa C40:3	106.5	105.0	111.9	114.2	115.7
PC aa C40:4	105.9	103.1	112.0	101.4	110.6
PC aa C40:5	106.8	104.8	113.8	104.3	108.7
PC aa C40:6	109.2	106.3	120.4	109.6	113.4
PC aa C42:0	109.2	107.5	114.2	80.3	105.5
PC aa C42:1	97.6	94.5	104.5	72.9	106.6
PC aa C42:2	105.3	96.3	105.7	103.7	107.5
PC aa C42:4	105.7	103.0	117.1	108.3	111.4
PC aa C42:5	108.3	105.9	117.6	102.7	108.8
PC aa C42:6	108.1	102.3	117.3	98.2	111.0
PC aa C44:0	103.3	107.6	110.1	95.0	106.2
PC aa C48:0	99.8	104.4	107.3	93.1	105.7

Figure S3. PCaa abundance patterns across CBD concentrations and incubation periods in wt and APPswe-expressing SH-SY5Y cells; wt 1 µM CBD/72 h is the reference dataset. Fold changes are relative to the corresponding control. Green indicates increases; red indicates decreases. Medium intensity denotes changes exceeding the mean standard error of the mean (mean SEM) as a descriptive guide, not a significance category. Dark color indicates nominal significance (unpaired Welch's t-test, $p \leq 0.05$). See Section 2.5.3 and Table S8 for methods and n. For wt 1 µM/72 h, BH-adjusted results are shown in Figure 1 and Table S7.

PCaa 0,25µM 24h

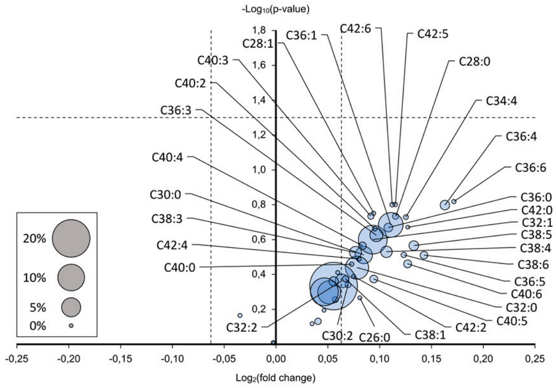

PCaa 0,25µM 72h

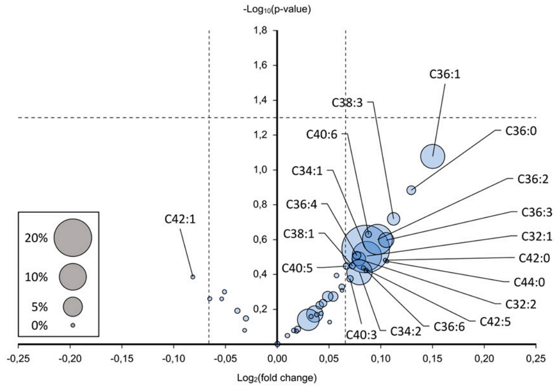

PCaa 1,0µM 24h

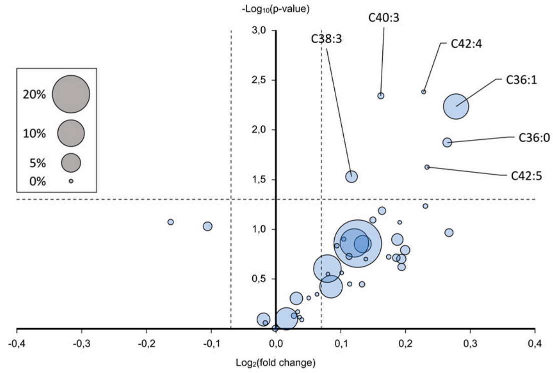

PCaa 1,0µM 72h

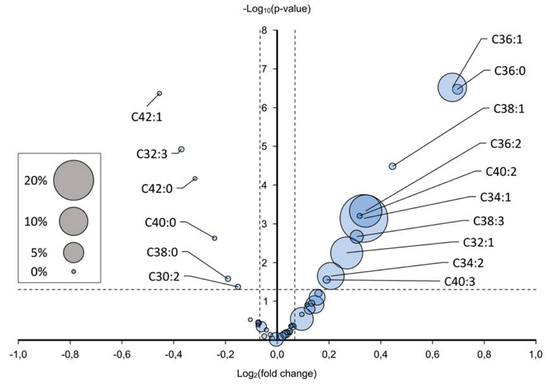

Figure S4. PCaa volcano plots for SH-SY5Y wt and APPswe-expressing cells versus matched solvent controls; exposures are indicated. Bubble area denotes each species' relative contribution to the measured PCaa class in CBD-treated cells; gray bubbles are size references. Vertical lines: mean SEM (descriptive); horizontal line: nominal p = 0.05 (Welch's t-test). See Section 2.5.3 and Table S8 for methods and n. Table S7 reports Welch's t-test p-values and, for wt 1 µM/72 h, BH-adjusted values corresponding to Figure 1.

PCaa 1,0µM 72h APPswe

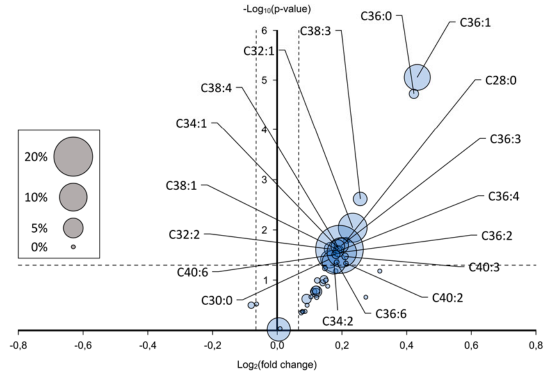

Species	SH-SY5Y-wt **0.25µM 24h**	SH-SY5Y-wt **0.25µM 72h**	SH-SY5Y-wt **1µM 24h**	SH-SY5Y-wt **1µM 72h**	SH-SY5Y- **APPswe** **1µM 72h**
PC ae C30:0	103.4	106.2	112.7	128.3	113.3
PC ae C30:1	106.1	103.4	114.1	120.4	117.7
PC ae C30:2	101.7	99.2	107.9	93.6	114.0
PC ae C32:1	107.1	109.6	112.7	137.7	124.7
PC ae C32:2	102.0	97.0	108.4	107.6	113.4
PC ae C34:0	108.0	114.4	114.5	151.7	112.4
PC ae C34:1	105.6	112.5	112.5	138.9	114.8
PC ae C34:2	106.7	107.4	110.7	127.4	118.1
PC ae C34:3	103.2	101.3	105.2	107.6	113.6
PC ae C36:0	96.8	98.1	103.2	133.1	120.0
PC ae C36:1	105.8	106.4	115.2	137.2	116.2
PC ae C36:2	102.4	102.5	106.4	121.2	113.2
PC ae C36:3	104.2	101.4	102.8	107.7	110.4
PC ae C36:4	105.9	103.1	106.5	98.5	100.8
PC ae C36:5	104.5	99.5	109.5	100.1	104.9
PC ae C38:0	107.2	100.7	111.0	111.3	122.7
PC ae C38:1	104.2	112.6	115.8	129.3	114.9
PC ae C38:2	106.9	103.7	110.3	118.6	111.5
PC ae C38:3	100.3	99.5	93.7	92.5	100.5
PC ae C38:4	99.9	96.0	95.6	89.3	104.4
PC ae C38:5	100.5	94.9	97.2	85.2	96.6
PC ae C38:6	102.9	97.7	103.5	92.2	101.1
PC ae C40:0	107.2	102.3	106.5	99.1	112.6
PC ae C40:1 / PC aa C40:8	113.1	107.3	122.0	112.9	112.6
PC ae C40:2	104.8	105.4	105.5	113.0	113.3
PC ae C40:3 / PC aa C40:10	102.6	95.0	98.1	93.0	101.7
PC ae C40:4	96.5	89.8	91.0	76.5	95.4
PC ae C40:5	97.5	90.3	96.2	77.1	97.8
PC ae C40:6	102.2	95.5	98.4	86.6	98.5
PC ae C42:0	103.5	101.2	111.6	98.4	109.2
PC ae C42:1	105.3	96.4	109.9	92.0	115.4
PC ae C42:2	106.9	97.4	107.5	95.7	108.1
PC ae C42:3	100.4	100.6	104.3	96.6	98.8
PC ae C42:4	103.8	103.1	110.5	94.9	102.9
PC ae C42:5	105.6	104.0	107.9	97.8	104.8
PC ae C44:3	101.5	83.0	104.9	86.9	114.0
PC ae C44:4	110.6	95.8	113.3	91.8	116.7
PC ae C44:5 / PC aa C44:12	103.0	103.2	113.8	97.0	108.4
PC ae C44:6	105.8	104.2	117.5	99.8	107.4

Figure S5. PCae abundance patterns across CBD concentrations and incubation periods in wt and APPswe-expressing SH-SY5Y cells; wt 1 µM CBD/72 h is the reference dataset. Fold changes are relative to the corresponding control. Green indicates increases; red indicates decreases. Medium intensity denotes changes exceeding the mean standard error of the mean (mean SEM) as a descriptive guide, not a significance category. Dark color indicates nominal significance (unpaired Welch's t-test, $p \leq 0.05$). See Section 2.5.3 and Table S8 for methods and n. For wt 1 µM/72 h, BH-adjusted results are shown in Figure 2 and Table S7.

PCae 0,25µM 24h

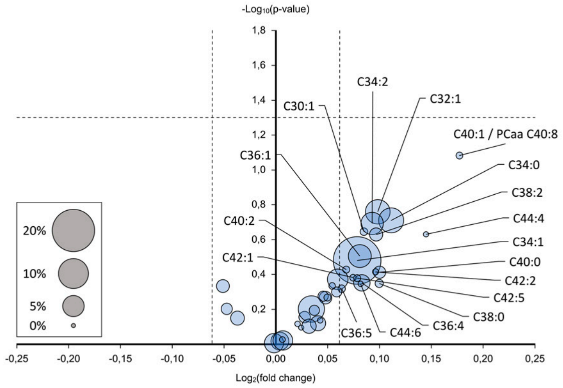

PCae 0,25µM 72h

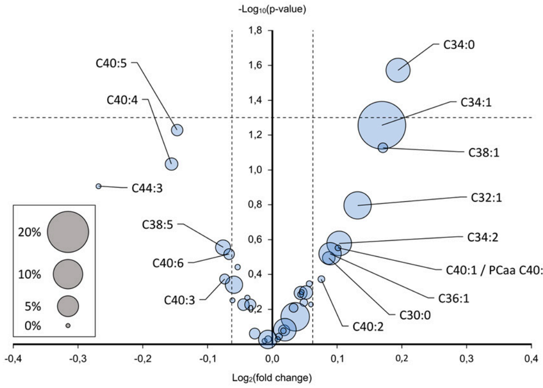

PCae 1,0µM 24h

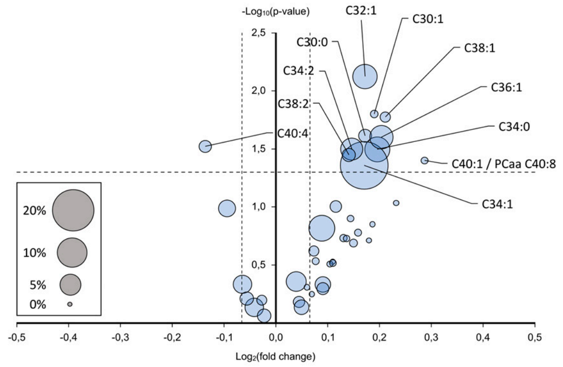

PCae 1,0µM 72h

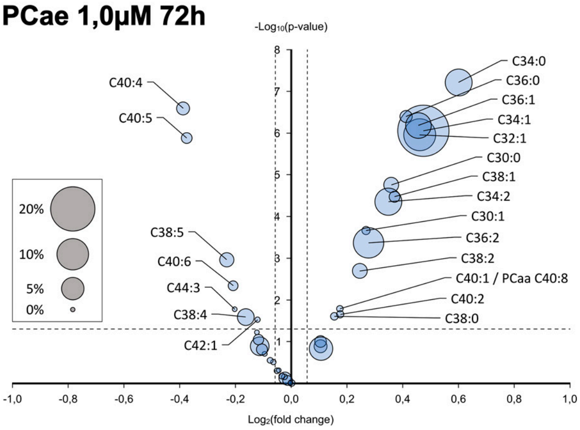

Figure S6. PCae volcano plots for SH-SY5Y wt and APPswe-expressing cells versus matched solvent controls; exposures are indicated. Bubble area denotes each species' relative contribution to the measured PCae class in CBD-treated cells; gray bubbles are size references. Vertical lines: mean SEM (descriptive); horizontal line: nominal p = 0.05 (Welch's t-test). See Section 2.5.3 and Table S8 for methods and n. Table S7 reports Welch's t-test p-values and, for wt 1 µM/72 h, BH-adjusted values corresponding to Figure 2.

PCae APPswe

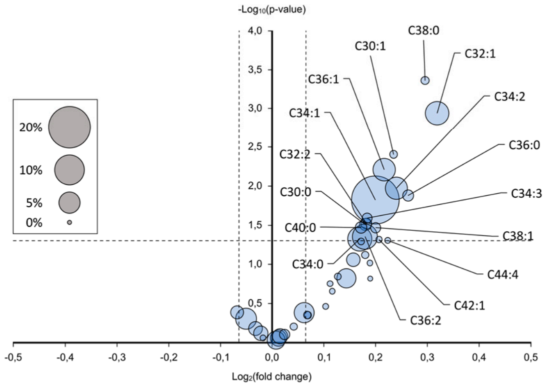

Table S1. Internal standards added before lipid extraction and used for normalization, their concentrations in the internal-standard mixture, and internal-standard detection limits based on calibration and blank measurements.

**Analyte group**	**Internal standard**	**Supplier and catalogue no.**	**Concentration in standard mixture (µM)**	**Detection limit (nM)***
PCaa – diacyl PC	06:0 PC (DHPC)	Avanti Polar Lipids, 850305P-200MG	3.33	13 ± 6
Pcae – nominally ether-containing PC	06:0 PC (DHPC)	Avanti Polar Lipids, 850305P-200MG	3.33	13 ± 6
LPC – lysophosphatidylcholines	19:0 Lyso PC	Avanti Polar Lipids, 855776P-25MG	3.33	18 ± 5
SM – sphingomyelins	06:0 SM (d18:1/6:0)	Avanti Polar Lipids, 860582C-5MG	4.72	23 ± 13
Free carnitine and acylcarnitines	Octanoyl-L-carnitine-d3<br>Palmitoyl-L-carnitine-d3	Supelco, 53230-10MG<br>Supelco, 55107-5MG	16.64<br>18.21	67 ± 12<br>136 ± 31

*Internal-standard detection limits were based on calibration and blank measurements and refer to the concentration in the cell-homogenate/internal-standard mixture used for extraction. For each standard, 14 concentration levels were prepared and 18 blank measurements were performed; the determination was conducted in three parallel extraction series, and values are mean ± SD. Free-carnitine and acylcarnitine signals were normalized to the arithmetic mean of the peak areas of both deuterated carnitine standards.

Table S2. Measurement parameters of the 4000-quadrupole linear-ion trap (Qtrap).

**Parameter**	**Used setting**
Measurement period	3 minutes
Scan type	MRM
Curtain gas	20 psi
Collision-induced dissociation	Medium
Ion spray voltage	5500 V
Temperature	200 °C
Ion source gas 1	40 psi
Ion source gas 2	50 psi
Interface heater	On
Entrance potential	10 V
Collision cell exit potential	15 V
Injection volume	20 µL
Draw speed	200 µL/min
Eject speed	200 µL/min
Needle cleaning	10 seconds

Table S3. Measurement parameters of the Orbitrap ID-X.

Parameter	Used setting
Ion source type	H-ESI
Spray voltage	Static
Positive Ion	3500 V
Gas Mode	Static
Sheath gas	10 Arb
Aux gas	5 Arb
Sweep gas	0 Arb
Ion transfer tube	300°C
Vaporizer temp	75°C
MS^n Level	2
Isolation mode	Quadrupole
Isolation window	1.6 m/z
Activation type	HCD
HCD collision energy type	Normalized
Detector type	Ion Trap
Ion trap scan rate	Rapid
Scan range mode	Auto
AGC target	Standard
Absolute AGC value	1.000e4
Maximum injection time mode	Auto
Microscans	1
Data type	Centroid
Polarity	Positive
Loop control	All
Time mode	Unscheduled

Table S4. MRM pairs of the detected lipid species in the 4000-quadrupole linear-ion trap (Qtrap).

Species	Q1 Mass (Da)	Q3 Mass (Da)
PC aa C20:0	566.2	184
PC aa C24:0	622.4	184
PC aa C26:0	650.5	184
PC aa C28:0	678.5	184
PC aa C28:1	676.5	184
PC aa C30:0	706.5	184
PC aa C30:2	702.5	184
PC aa C32:0	734.6	184
PC aa C32:1	732.6	184
PC aa C32:2	730.5	184
PC aa C32:3	728.5	184
PC aa C34:1	760.6	184
PC aa C34:2	758.6	184
PC aa C34:3	756.6	184
PC aa C34:4	754.5	184
PC aa C36:0	790.6	184
PC aa C36:1	788.6	184
PC aa C36:2	786.6	184
PC aa C36:3	784.6	184
PC aa C36:4	782.6	184
PC aa C36:5	780.6	184
PC aa C36:6	778.5	184
PC aa C38:0	818.7	184
PC aa C38:1	816.7	184
PC aa C38:3	812.6	184
PC aa C38:4	810.6	184
PC aa C38:5	808.6	184
PC aa C38:6	806.6	184
PC aa C40:0	846.7	184
PC aa C40:1	844.7	184

PC aa C40:2	842.7	184
PC aa C40:3	840.7	184
PC aa C40:4	838.6	184
PC aa C40:5	836.6	184
PC aa C40:6	834.6	184
PC aa C42:0	874.7	184
PC aa C42:1	872.7	184
PC aa C42:2	870.7	184
PC aa C42:4	866.7	184
PC aa C42:5	864.7	184
PC aa C42:6	862.6	184
PC aa C44:0	902.7	184
PC aa C48:0	958.8	184
PC aa Std 06:0 PC DHPC	454.4	184
PC ae C30:0	692.6	184
PC ae C30:1	690.5	184
PC ae C30:2	688.5	184
PC ae C32:1	718.6	184
PC ae C32:2	716.6	184
PC ae C34:0	748.6	184
PC ae C34:1	746.6	184
PC ae C34:2	744.6	184
PC ae C34:3	742.6	184
PC ae C36:0	776.7	184
PC ae C36:1	774.6	184
PC ae C36:2	772.6	184
PC ae C36:3	770.6	184
PC ae C36:4	768.6	184
PC ae C36:5	766.6	184
PC ae C38:0	804.7	184
PC ae C38:1	802.7	184
PC ae C38:2	800.7	184
PC ae C38:3	798.6	184
PC ae C38:4	796.6	184
PC ae C38:5	794.6	184
PC ae C38:6	792.6	184
PC ae C40:0	832.7	184
PC ae C40:1 / PC aa C40:8	830.7	184
PC ae C40:2	828.7	184
PC ae C40:3 / PC aa C40:10	826.7	184
PC ae C40:4	824.7	184
PC ae C40:5	822.6	184
PC ae C40:6	820.6	184
PC ae C42:0	860.8	184
PC ae C42:1	858.7	184
PC ae C42:2	856.7	184
PC ae C42:3	854.7	184
PC ae C42:4	852.7	184
PC ae C42:5	850.7	184
PC ae C44:3	882.7	184
PC ae C44:4	880.7	184
PC ae C44:5 / PC aa C44:12	878.7	184
PC ae C44:6	876.7	184
Lyso PC 06:0	356.2	184
Lyso PC 10:0	412.3	184
Lyso PC 12:0	440.4	184
Lyso PC 14:0	468.3	184
Lyso PC 16:0	496.3	184
Lyso PC 16:1	494.3	184
Lyso PC 17:0	510.3	184
Lyso PC 18:0	524.3	184
Lyso PC 18:1	522.3	184

Lyso PC 18:2	520.3	184
Lyso PC 18:3	518.4	184
Lyso PC 20:0	552.4	184
Lyso PC 20:3	546.3	184
Lyso PC 20:4	544.3	184
Lyso PC 20:5	542.4	184
Lyso PC 22:0	580.5	184
Lyso PC 22:6	568.4	184
Lyso PC 24:0	608.4	184
Lyso PC 26:0	636.5	184
Lyso PC 26:1	634.4	184
Lyso PC 28:0	664.5	184
Lyso PC 28:1	662.5	184
Lyso PC Std 19:0	538.6	184
SM C14:1 OH	689.6	184
SM C16:0	703.6	184
SM C16:1	701.6	184
SM C16:1 OH	717.6	184
SM C18:0	731.6	184
SM C18:1	729.6	184
SM C20:2	755.6	184
SM C22:1 OH	801.7	184
SM C22:2 OH	799.7	184
SM C24:0	815.7	184
SM C24:1	813.7	184
SM C24:1 OH	829.7	184
SM C26:0	843.7	184
SM C26:1	841.7	184
SM Std d18:1/6:0	563.6	184
C00	162.1	85.1
C02	204.1	85.1
C03	218.1	85.1
C03 OH	234.1	85.1
C03:1	216.1	85.1
C04	232.2	85.1
C04 OH	248.1	85.1
C04:1	230.1	85.1
C05	246.2	85.1
C05 M DC	290.2	85.1
C05 OH	262.2	85.1
C05:1	244.2	85.1
C05:1 DC	274.1	85.1
C06	260.2	85.1
C06 OH	276.2	85.1
C06:1	258.2	85.1
C07 DC	304.2	85.1
C08	288.2	85.1
C08:1	286.2	85.1
C09	302.2	85.1
C10	316.2	85.1
C10:1	314.2	85.1
C10:2	312.2	85.1
C12	344.3	85.1
C12 DC	374.3	85.1
C12:1	342.3	85.1
C14	372.3	85.1
C14:1	370.3	85.1
C14:1 OH	386.3	85.1
C14:2	368.3	85.1
C14:2 OH	384.3	85.1
C16	400.3	85.1
C16 OH	416.3	85.1

C16:1	398.3	85.1
C16:1 OH	414.3	85.1
C16:2	396.3	85.1
C16:2 OH	412.3	85.1
C18:0	428.4	85.1
C18:1	426.4	85.1
C18:1 OH	442.4	85.1
C18:2	424.3	85.1
CSTD 08:0 D3	291.2	85.1
CSTD 16:0 D3	403.3	85.1

Table S5. Monitored precursor/product-ion pairs of the detected lipid species in the Orbitrap ID-X.

Species	Precursor m/z	Product m/z
PC aa C20:0	566.3816	184.0733
PC aa C24:0	622.4442	184.0733
PC aa C26:0	650.4755	184.0733
PC aa C28:0	678.5068	184.0733
PC aa C28:1	676.4912	184.0733
PC aa C30:0	706.5381	184.0733
PC aa C30:2	702.5068	184.0733
PC aa C32:0	734.5694	184.0733
PC aa C32:1	732.5538	184.0733
PC aa C32:2	730.5381	184.0733
PC aa C32:3	728.5225	184.0733
PC aa C34:1	760.5851	184.0733
PC aa C34:2	758.5694	184.0733
PC aa C34:3	756.5538	184.0733
PC aa C34:4	754.5381	184.0733
PC aa C36:0	790.632	184.0733
PC aa C36:1	788.6164	184.0733
PC aa C36:2	786.6007	184.0733
PC aa C36:3	784.5851	184.0733
PC aa C36:4	782.5694	184.0733
PC aa C36:5	780.5538	184.0733
PC aa C36:6	778.5381	184.0733
PC aa C38:0	818.6633	184.0733
PC aa C38:1	816.6477	184.0733
PC aa C38:3	812.6164	184.0733
PC aa C38:4	810.6007	184.0733
PC aa C38:5	808.5851	184.0733
PC aa C38:6	806.5694	184.0733
PC aa C40:0	846.6946	184.0733
PC aa C40:1	844.679	184.0733
PC aa C40:2	842.6633	184.0733
PC aa C40:3	840.6477	184.0733
PC aa C40:4	838.632	184.0733
PC aa C40:5	836.6164	184.0733
PC aa C40:6	834.6007	184.0733
PC aa C42:0	874.7259	184.0733
PC aa C42:1	872.7103	184.0733
PC aa C42:2	870.6946	184.0733
PC aa C42:4	866.6633	184.0733
PC aa C42:5	864.6477	184.0733
PC aa C42:6	862.632	184.0733
PC aa C44:0	902.7572	184.0733
PC aa C48:0	958.8198	184.0733
PC aa Std 06:0 PC DHPC	454.2564	184.0733
PC ae C30:0	692.5589	184.0733
PC ae C30:1	690.5432	184.0733
PC ae C30:2	688.5276	184.0733

PC ae C32:1	718.5745	184.0733
PC ae C32:2	716.5589	184.0733
PC ae C34:0	748.6215	184.0733
PC ae C34:1	746.6058	184.0733
PC ae C34:2	744.5902	184.0733
PC ae C34:3	742.5745	184.0733
PC ae C36:0	776.6528	184.0733
PC ae C36:1	774.6371	184.0733
PC ae C36:2	772.6215	184.0733
PC ae C36:3	770.6058	184.0733
PC ae C36:4	768.5902	184.0733
PC ae C36:5	766.5745	184.0733
PC ae C38:0	804.6841	184.0733
PC ae C38:1	802.6684	184.0733
PC ae C38:2	800.6528	184.0733
PC ae C38:3	798.6371	184.0733
PC ae C38:4	796.6215	184.0733
PC ae C38:5	794.6058	184.0733
PC ae C38:6	792.5902	184.0733
PC ae C40:0	832.7154	184.0733
PC ae C40:1 / PC aa C40:8	830.6997	184.0733
PC ae C40:2	828.6841	184.0733
PC ae C40:3 / PC aa C40:10	826.6684	184.0733
PC ae C40:4	824.6528	184.0733
PC ae C40:5	822.6371	184.0733
PC ae C40:6	820.6215	184.0733
PC ae C42:0	860.7467	184.0733
PC ae C42:1	858.731	184.0733
PC ae C42:2	856.7154	184.0733
PC ae C42:3	854.6997	184.0733
PC ae C42:4	852.6841	184.0733
PC ae C42:5	850.6684	184.0733
PC ae C44:3	882.731	184.0733
PC ae C44:4	880.7154	184.0733
PC ae C44:5 / PC aa C44:12	878.6997	184.0733
PC ae C44:6	876.6841	184.0733
Lyso PC 06:0	356.1833	184.0733
Lyso PC 10:0	412.2459	184.0733
Lyso PC 12:0	440.2772	184.0733
Lyso PC 14:0	468.3085	184.0733
Lyso PC 16:0	496.3398	184.0733
Lyso PC 16:1	494.3241	184.0733
Lyso PC 17:0	510.3554	184.0733
Lyso PC 18:0	524.3711	184.0733
Lyso PC 18:1	522.3554	184.0733
Lyso PC 18:2	520.3398	184.0733
Lyso PC 18:3	518.3241	184.0733
Lyso PC 20:0	552.4024	184.0733
Lyso PC 20:3	546.3554	184.0733
Lyso PC 20:4	544.3398	184.0733
Lyso PC 20:5	542.3241	184.0733
Lyso PC 22:0	580.4337	184.0733
Lyso PC 22:6	568.3398	184.0733
Lyso PC 24:0	608.465	184.0733
Lyso PC 26:0	636.4963	184.0733
Lyso PC 26:1	634.4806	184.0733
Lyso PC 28:0	664.5276	184.0733
Lyso PC 28:1	662.5119	184.0733
Lyso PC Std 19:0	538.3867	184.0733
SM C14:1 OH	689.5228	184.0733
SM C16:0	703.5749	184.0733
SM C16:1	701.5592	184.0733

SM C16:1 OH	717.5541	184.0733
SM C18:0	731.6062	184.0733
SM C18:1	729.5905	184.0733
SM C20:2	755.6062	184.0733
SM C22:1 OH	801.648	184.0733
SM C22:2 OH	799.6324	184.0733
SM C24:0	815.7001	184.0733
SM C24:1	813.6844	184.0733
SM C24:1 OH	829.6793	184.0733
SM C26:0	843.7314	184.0733
SM C26:1	841.7157	184.0733
SM Std d18:1/6:0	563.4184	184.0733
C00	162.1125	85.0295
C02	204.123	85.0295
C03	218.1387	85.0295
C03 OH	234.1336	85.0295
C03:1	216.123	85.0295
C04	232.1543	85.0295
C04 OH	248.1492	85.0295
C04:1	230.1387	85.0295
C05	246.170	85.0295
C05 M DC	290.1598	85.0295
C05 OH	262.1649	85.0295
C05:1	244.1543	85.0295
C05:1 DC	274.1285	85.0295
C06	260.1856	85.0295
C06 OH	276.1805	85.0295
C06:1	258.170	85.0295
C07 DC	304.1755	85.0295
C08	288.2169	85.0295
C08:1	286.2013	85.0295
C09	302.2326	85.0295
C10	316.2482	85.0295
C10:1	314.2326	85.0295
C10:2	312.2169	85.0295
C12	344.2795	85.0295
C12 DC	374.2537	85.0295
C12:1	342.2639	85.0295
C14	372.3108	85.0295
C14:1	370.2952	85.0295
C14:1 OH	386.2901	85.0295
C14:2	368.2795	85.0295
C14:2 OH	384.2744	85.0295
C16	400.3421	85.0295
C16 OH	416.337	85.0295
C16:1	398.3265	85.0295
C16:1 OH	414.3214	85.0295
C16:2	396.3108	85.0295
C16:2 OH	412.3057	85.0295
C18:0	428.3734	85.0295
C18:1	426.3578	85.0295
C18:1 OH	442.3527	85.0295
C18:2	424.3421	85.0295
CSTD 08:0 D3	291.2358	85.0295
CSTD 16:0 D3	403.361	85.0295

Table S6. Overview of the lipid and carnitine-related analyte groups covered by the targeted analytical panel.

Analyte group	Number of monitored analytes
Diacyl phosphatidylcholine sum species (Pcaa)	43
Assay-specific Pcae signals	39
Lysophosphatidylcholines (LPC)	22
Sphingomyelins (SM)	14
Carnitine-related analytes, including free carnitine and acylcarnitines	41
**Total**	**159**

Table S7. Welch's t-test p-values and Benjamini–Hochberg (BH)-adjusted p-values (q) for the wt 1 µM/72 h reference dataset, and Welch's t-test p-values for the additional PCaa and PCae conditions. BH adjustment was applied separately to individual analytes within each biochemical class and to grouped endpoints within each lipid-class-specific saturation-class, double-bond-count, chain-length-class, or chain-length set. The PCaa chain-length family comprises 15 comparisons, including the <C30:X and >C42:X groups. Here, p denotes the unadjusted Welch's t-test p-value and q the BH-adjusted p-value. Asterisks refer to the value in the respective column: * ≤ 0.05, ** ≤ 0.01, *** ≤ 0.001; ns, non-significant.

**SH-SY5Y wt, 1 µM CBD, 72 h**		
**Species**	**p (Welch's t-test)**	**q (BH-adjusted)**
PC aa C20:0	0.424203 (ns)	0.613091 (ns)
PC aa C24:0	0.919235 (ns)	0.941121 (ns)
PC aa C26:0	0.800984 (ns)	0.850677 (ns)
PC aa C28:0	0.062299 (ns)	0.157581 (ns)
PC aa C28:1	0.641202 (ns)	0.765880 (ns)
PC aa C30:0	0.120829 (ns)	0.251335 (ns)
PC aa C30:2	0.042481 (*)	0.114168 (ns)
PC aa C32:0	0.286429 (ns)	0.513134 (ns)
PC aa C32:1	0.005607 (**)	0.020093 (*)
PC aa C32:2	0.964256 (ns)	0.964256 (ns)
PC aa C32:3	0.000012 (***)	0.000128 (***)
PC aa C34:1	0.000723 (***)	0.003453 (**)
PC aa C34:2	0.022298 (*)	0.073756 (ns)
PC aa C34:3	0.456253 (ns)	0.613091 (ns)
PC aa C34:4	0.366593 (ns)	0.583833 (ns)
PC aa C36:0	<0.000001 (***)	0.000006 (***)
PC aa C36:1	<0.000001 (***)	0.000006 (***)
PC aa C36:2	0.000465 (***)	0.002858 (**)
PC aa C36:3	0.078135 (ns)	0.186657 (ns)
PC aa C36:4	0.684512 (ns)	0.795513 (ns)
PC aa C36:5	0.352925 (ns)	0.583684 (ns)
PC aa C36:6	0.218768 (ns)	0.409002 (ns)
PC aa C38:0	0.026402 (*)	0.079716 (ns)
PC aa C38:1	0.000033 (***)	0.000283 (***)
PC aa C38:3	0.002146 (**)	0.009155 (**)
PC aa C38:4	0.157509 (ns)	0.307858 (ns)
PC aa C38:5	0.716862 (ns)	0.804472 (ns)
PC aa C38:6	0.593639 (ns)	0.750779 (ns)
PC aa C40:0	0.002342 (**)	0.009155 (**)
PC aa C40:1	0.549557 (ns)	0.716090 (ns)
PC aa C40:2	0.000623 (***)	0.003350 (**)
PC aa C40:3	0.027808 (*)	0.079716 (ns)
PC aa C40:4	0.811111 (ns)	0.850677 (ns)
PC aa C40:5	0.449870 (ns)	0.613091 (ns)
PC aa C40:6	0.112179 (ns)	0.251335 (ns)
PC aa C42:0	0.000068 (***)	0.000486 (***)
PC aa C42:1	<0.000001 (***)	0.000006 (***)
PC aa C42:2	0.438367 (ns)	0.613091 (ns)
PC aa C42:4	0.122745 (ns)	0.251335 (ns)
PC aa C42:5	0.636319 (ns)	0.765880 (ns)
PC aa C42:6	0.729638 (ns)	0.804472 (ns)

SH-SY5Y wt, 1 µM CBD, 72 h

Species	p (Welch's t-test)	q (BH-adjusted)
PC aa C44:0	0.410557 (ns)	0.613091 (ns)
PC aa C48:0	0.298334 (ns)	0.513134 (ns)
PC ae C30:0	0.000018 (***)	0.000085 (***)
PC ae C30:1	0.000216 (***)	0.000766 (***)
PC ae C30:2	0.196014 (ns)	0.263605 (ns)
PC ae C32:1	0.000001 (***)	0.000007 (***)
PC ae C32:2	0.129991 (ns)	0.194986 (ns)
PC ae C34:0	<0.000001 (***)	0.000002 (***)
PC ae C34:1	<0.000001 (***)	0.000007 (***)
PC ae C34:2	0.000044 (***)	0.000172 (***)
PC ae C34:3	0.099028 (ns)	0.160921 (ns)
PC ae C36:0	<0.000001 (***)	0.000005 (***)
PC ae C36:1	<0.000001 (***)	0.000006 (***)
PC ae C36:2	0.000430 (***)	0.001399 (**)
PC ae C36:3	0.147961 (ns)	0.213721 (ns)
PC ae C36:4	0.754154 (ns)	0.817001 (ns)
PC ae C36:5	0.978440 (ns)	0.978638 (ns)
PC ae C38:0	0.024798 (*)	0.050352 (ns)
PC ae C38:1	0.000034 (***)	0.000145 (***)
PC ae C38:2	0.002015 (**)	0.005613 (**)
PC ae C38:3	0.129489 (ns)	0.194986 (ns)
PC ae C38:4	0.025821 (*)	0.050352 (ns)
PC ae C38:5	0.001087 (**)	0.003261 (**)
PC ae C38:6	0.088713 (ns)	0.150427 (ns)
PC ae C40:0	0.850143 (ns)	0.896097 (ns)
PC ae C40:1 / PC aa C40:8	0.016227 (*)	0.038603 (*)
PC ae C40:2	0.022233 (*)	0.048171 (*)
PC ae C40:3 / PC aa C40:10	0.154360 (ns)	0.215002 (ns)
PC ae C40:4	<0.000001 (***)	0.000005 (***)
PC ae C40:5	0.000001 (***)	0.000007 (***)
PC ae C40:6	0.004577 (**)	0.011901 (*)
PC ae C42:0	0.697176 (ns)	0.776853 (ns)
PC ae C42:1	0.029900 (*)	0.055529 (ns)
PC ae C42:2	0.307887 (ns)	0.387342 (ns)
PC ae C42:3	0.498713 (ns)	0.589388 (ns)
PC ae C42:4	0.280531 (ns)	0.364690 (ns)
PC ae C42:5	0.671712 (ns)	0.770493 (ns)
PC ae C44:3	0.016827 (*)	0.038603 (*)
PC ae C44:4	0.059790 (ns)	0.105991 (ns)
PC ae C44:5 / PC aa C44:12	0.486973 (ns)	0.589388 (ns)
PC ae C44:6	0.978638 (ns)	0.978638 (ns)
Lyso PC C06:0	0.829108 (ns)	0.829108 (ns)
Lyso PC C10:0	0.015251 (*)	0.026947 (*)
Lyso PC C12:0	0.326081 (ns)	0.358689 (ns)
Lyso PC C14:0	0.000392 (***)	0.001726 (**)
Lyso PC C16:0	0.015923 (*)	0.026947 (*)
Lyso PC C16:1	0.000545 (***)	0.001997 (**)
Lyso PC C17:0	0.003576 (**)	0.008740 (**)
Lyso PC C18:0	0.229442 (ns)	0.265670 (ns)
Lyso PC C18:1	0.013531 (*)	0.026947 (*)
Lyso PC C18:2	0.000272 (***)	0.001495 (**)
Lyso PC C18:3	0.000795 (***)	0.002498 (**)
Lyso PC C20:0	0.014146 (*)	0.026947 (*)
Lyso PC C20:3	<0.000001 (***)	0.000004 (***)
Lyso PC C20:4	<0.000001 (***)	<0.000001 (***)
Lyso PC C20:5	0.024730 (*)	0.036271 (*)
Lyso PC C22:0	0.002890 (**)	0.007946 (**)
Lyso PC C22:6	<0.000001 (***)	0.000003 (***)
Lyso PC C24:0	0.045977 (*)	0.063219 (ns)
Lyso PC C26:0	0.186528 (ns)	0.227979 (ns)

SH-SY5Y wt, 1 µM CBD, 72 h

Species	p (Welch's t-test)	q (BH-adjusted)
Lyso PC C26:1	0.023701 (*)	0.036271 (*)
Lyso PC C28:0	0.811950 (ns)	0.829108 (ns)
Lyso PC C28:1	0.051316 (ns)	0.066409 (ns)
SM C14:1 OH	0.349791 (ns)	0.445189 (ns)
SM C16:0	0.003328 (**)	0.007765 (**)
SM C16:1	0.222691 (ns)	0.346409 (ns)
SM C16:1 OH	0.007136 (**)	0.014272 (*)
SM C18:0	0.000060 (***)	0.000168 (***)
SM C18:1	<0.000001 (***)	<0.000001 (***)
SM C20:2	<0.000001 (***)	0.000002 (***)
SM C22:1 OH	0.730199 (ns)	0.730199 (ns)
SM C22:2 OH	<0.000001 (***)	<0.000001 (***)
SM C24:0	0.000022 (***)	0.000079 (***)
SM C24:1	0.584016 (ns)	0.645161 (ns)
SM C24:1 OH	0.599078 (ns)	0.645161 (ns)
SM C26:0	0.247439 (ns)	0.346415 (ns)
SM C26:1	0.156273 (ns)	0.273479 (ns)
C0	<0.000001 (***)	0.000004 (***)
C02	<0.000001 (***)	0.000005 (***)
C03	<0.000001 (***)	<0.000001 (***)
C03 OH	0.000002 (***)	0.000012 (***)
C03:1	0.000555 (***)	0.001518 (**)
C04	<0.000001 (***)	<0.000001 (***)
C04 OH / C3 DC	0.001101 (**)	0.002654 (**)
C04:1	0.009347 (**)	0.013687 (*)
C05	<0.000001 (***)	<0.000001 (***)
C05 M DC	0.920384 (ns)	0.920384 (ns)
C05 OH / C3 M DC	0.000007 (***)	0.000033 (***)
C05:1	0.009111 (**)	0.013687 (*)
C05:1 DC	0.000826 (***)	0.002115 (**)
C06 / C4:1 DC	0.003113 (**)	0.006079 (**)
C06 OH / C5 DC	0.559401 (ns)	0.588089 (ns)
C06:1	<0.000001 (***)	<0.000001 (***)
C07 DC	0.000020 (***)	0.000073 (***)
C08	0.311553 (ns)	0.354824 (ns)
C08:1	0.000194 (***)	0.000612 (***)
C09	0.002953 (**)	0.006054 (**)
C10	0.008710 (**)	0.013687 (*)
C10:1	0.005077 (**)	0.009050 (**)
C10:2	0.290661 (ns)	0.340489 (ns)
C12	0.000146 (***)	0.000498 (***)
C12 DC	0.199994 (ns)	0.241169 (ns)
C12:1	0.001727 (**)	0.003727 (**)
C14	0.000409 (***)	0.001198 (**)
C14:1	0.008422 (**)	0.013687 (*)
C14:1 OH	0.068001 (ns)	0.089937 (ns)
C14:2	0.067042 (ns)	0.089937 (ns)
C14:2 OH	0.004936 (**)	0.009050 (**)
C16	0.675073 (ns)	0.691950 (ns)
C16 OH	0.557353 (ns)	0.588089 (ns)
C16:1	<0.000001 (***)	<0.000001 (***)
C16:1 OH	0.030841 (*)	0.043602 (*)
C16:2	0.005410 (**)	0.009242 (**)
C16:2 OH	0.476744 (ns)	0.528284 (ns)
C18:0	0.142302 (ns)	0.176800 (ns)
C18:1	0.000019 (***)	0.000073 (***)
C18:1 OH	0.130894 (ns)	0.167708 (ns)
C18:2	0.001400 (**)	0.003189 (**)

SH-SY5Y wt, 1 µM CBD, 72 h

Parameter	p (Welch's t-test)	q (BH-adjusted)
PC aa SFA	0.094467 (ns)	0.094467 (ns)
PC aa MUFA	0.000240 (***)	0.000719 (***)
PC aa PUFA	0.019432 (*)	0.029148 (*)
PC aa CX:0	0.094467 (ns)	0.165318 (ns)
PC aa CX:1	0.000240 (***)	0.001678 (**)
PC aa CX:2	0.005778 (**)	0.020222 (*)
PC aa CX:3	0.092327 (ns)	0.165318 (ns)
PC aa CX:4	0.411533 (ns)	0.480122 (ns)
PC aa CX:5	0.729549 (ns)	0.729549 (ns)
PC aa CX:6	0.331915 (ns)	0.464681 (ns)
PC aa short-chain (< C32:X)	0.144415 (ns)	0.144415 (ns)
PC aa medium-chain (C32:X - C36:X)	0.001563 (**)	0.004688 (**)
PC aa long-chain (> C36:X)	0.065019 (ns)	0.097528 (ns)
PC aa C20:X	0.424203 (ns)	0.530254 (ns)
PC aa C24:X	0.919235 (ns)	0.919235 (ns)
PC aa C26:X	0.800984 (ns)	0.904905 (ns)
PC aa C28:X	0.107356 (ns)	0.322069 (ns)
PC aa <C30:X	0.322432 (ns)	0.483647 (ns)
PC aa C30:X	0.150286 (ns)	0.375715 (ns)
PC aa C32:X	0.049244 (*)	0.184667 (ns)
PC aa C34:X	0.002352 (**)	0.017639 (*)
PC aa C36:X	0.000041 (***)	0.000613 (***)
PC aa C38:X	0.030127 (*)	0.150634 (ns)
PC aa C40:X	0.216969 (ns)	0.464934 (ns)
PC aa C42:X	0.844578 (ns)	0.904905 (ns)
PC aa >C42:X	0.320652 (ns)	0.483647 (ns)
PC aa C44:X	0.410557 (ns)	0.530254 (ns)
PC aa C48:X	0.298334 (ns)	0.483647 (ns)
PC ae SFA	<0.000001 (***)	0.000002 (***)
PC ae MUFA	0.000001 (***)	0.000002 (***)
PC ae PUFA	0.300088 (ns)	0.300088 (ns)
PC ae CX:0	<0.000001 (***)	0.000004 (***)
PC ae CX:1	0.000001 (***)	0.000004 (***)
PC ae CX:2	0.000393 (***)	0.000729 (***)
PC ae CX:3	0.840562 (ns)	0.840562 (ns)
PC ae CX:4	0.006528 (**)	0.009140 (**)
PC ae CX:5	0.000417 (***)	0.000729 (***)
PC ae CX:6	0.025405 (*)	0.029639 (*)
PC ae short-chain (< C32:X)	0.000031 (***)	0.000047 (***)
PC ae medium-chain (C32 - C36:X)	0.000009 (***)	0.000027 (***)
PC ae long-chain (> C36:X)	0.141698 (ns)	0.141698 (ns)
PC ae C30:X	0.000031 (***)	0.000084 (***)
PC ae C32:X	0.000005 (***)	0.000021 (***)
PC ae C34:X	0.000001 (***)	0.000010 (***)
PC ae C36:X	0.000581 (***)	0.001162 (**)
PC ae C38:X	0.495498 (ns)	0.495498 (ns)
PC ae C40:X	0.002174 (**)	0.003479 (**)
PC ae C42:X	0.368318 (ns)	0.420935 (ns)
PC ae > C42:X	0.104113 (ns)	0.138818 (ns)
Lyso PC SFA	0.042703 (*)	0.042703 (*)
Lyso PC MUFA	0.002910 (**)	0.004365 (**)

SH-SY5Y wt, 1 µM CBD, 72 h		
**Parameter**	**p (Welch's t-test)**	**q (BH-adjusted)**
Lyso PC PUFA	0.000001 (***)	0.000004 (***)
Lyso PC short-chain (< C16:X)	0.410247 (ns)	0.410247 (ns)
Lyso PC medium-chain (C16 - C20X)	0.008664 (**)	0.025991 (*)
Lyso PC long-chain (> C20:X)	0.070686 (ns)	0.106029 (ns)
SM SFA	0.017773 (*)	0.026659 (*)
SM MUFA	0.531500 (ns)	0.531500 (ns)
SM PUFA	<0.000001 (***)	<0.000001 (***)
SM C14:X	0.349791 (ns)	0.393515 (ns)
SM C16:X	0.004343 (**)	0.009772 (**)
SM C16:X OH	0.007136 (**)	0.012845 (*)
SM C18:X	0.000020 (***)	0.000091 (***)
SM C20:X	<0.000001 (***)	0.000003 (***)
SM C22:X OH	0.000035 (***)	0.000106 (***)
SM C24:X	0.083340 (ns)	0.125010 (ns)
SM C24:X OH	0.599078 (ns)	0.599078 (ns)
SM C26:X	0.331483 (ns)	0.393515 (ns)

**Species**	**wt 0.25 µM CBD, 24 h**<br>**p (Welch's t-test)**	**wt 1 µM CBD, 24 h**<br>**p (Welch's t-test)**
PC aa C20:0	0.7632 (ns)	0.4899 (ns)
PC aa C24:0	0.9876 (ns)	0.7647 (ns)
PC aa C26:0	0.5410 (ns)	0.8087 (ns)
PC aa C28:0	0.1844 (ns)	0.0806 (ns)
PC aa C28:1	0.1773 (ns)	0.1255 (ns)
PC aa C30:0	0.3074 (ns)	0.1406 (ns)
PC aa C30:2	0.4568 (ns)	0.7386 (ns)
PC aa C32:0	0.3636 (ns)	0.3778 (ns)
PC aa C32:1	0.2515 (ns)	0.1369 (ns)
PC aa C32:2	0.4330 (ns)	0.8088 (ns)
PC aa C32:3	0.5550 (ns)	0.0846 (ns)
PC aa C34:1	0.4642 (ns)	0.1389 (ns)
PC aa C34:2	0.5044 (ns)	0.7931 (ns)
PC aa C34:3	0.4352 (ns)	0.0933 (ns)
PC aa C34:4	0.1866 (ns)	0.8713 (ns)
PC aa C36:0	0.2141 (ns)	0.0134 (*)
PC aa C36:1	0.2067 (ns)	0.0058 (**)
PC aa C36:2	0.5010 (ns)	0.2480 (ns)
PC aa C36:3	0.2364 (ns)	0.4956 (ns)
PC aa C36:4	0.1592 (ns)	0.1613 (ns)
PC aa C36:5	0.3072 (ns)	0.3564 (ns)
PC aa C36:6	0.1518 (ns)	0.1888 (ns)
PC aa C38:0	0.7393 (ns)	0.9926 (ns)
PC aa C38:1	0.4220 (ns)	0.1871 (ns)
PC aa C38:3	0.2974 (ns)	0.0296 (*)
PC aa C38:4	0.2947 (ns)	0.1266 (ns)
PC aa C38:5	0.2718 (ns)	0.1984 (ns)
PC aa C38:6	0.3088 (ns)	0.2389 (ns)
PC aa C40:0	0.3477 (ns)	0.3542 (ns)
PC aa C40:1	0.3883 (ns)	0.6736 (ns)
PC aa C40:2	0.2173 (ns)	0.1458 (ns)
PC aa C40:3	0.1841 (ns)	0.0045 (**)
PC aa C40:4	0.2720 (ns)	0.0649 (ns)
PC aa C40:5	0.4224 (ns)	0.1934 (ns)
PC aa C40:6	0.3454 (ns)	0.1080 (ns)
PC aa C42:0	0.2129 (ns)	0.0850 (ns)
PC aa C42:1	0.6840 (ns)	0.4498 (ns)

Species	wt 0.25 µM CBD, 24 h<br>p (Welch's t-test)	wt 1 µM CBD, 24 h<br>p (Welch's t-test)
PC aa C42:2	0.4080 (ns)	0.2817 (ns)
PC aa C42:4	0.3242 (ns)	0.0042 (**)
PC aa C42:5	0.1579 (ns)	0.0237 (*)
PC aa C42:6	0.1580 (ns)	0.0582 (ns)
PC aa C44:0	0.6389 (ns)	0.1987 (ns)
PC aa C48:0	0.9762 (ns)	0.2747 (ns)
PC ae C30:0	0.5410 (ns)	0.0243 (*)
PC ae C30:1	0.2270 (ns)	0.0171 (*)
PC ae C30:2	0.8035 (ns)	0.2967 (ns)
PC ae C32:1	0.1742 (ns)	0.0076 (**)
PC ae C32:2	0.6984 (ns)	0.0992 (ns)
PC ae C34:0	0.1949 (ns)	0.0321 (*)
PC ae C34:1	0.3308 (ns)	0.0438 (*)
PC ae C34:2	0.2035 (ns)	0.0317 (*)
PC ae C34:3	0.5299 (ns)	0.2394 (ns)
PC ae C36:0	0.6266 (ns)	0.6581 (ns)
PC ae C36:1	0.3118 (ns)	0.0254 (*)
PC ae C36:2	0.6285 (ns)	0.1527 (ns)
PC ae C36:3	0.4251 (ns)	0.4401 (ns)
PC ae C36:4	0.4448 (ns)	0.4654 (ns)
PC ae C36:5	0.4803 (ns)	0.1849 (ns)
PC ae C38:0	0.4497 (ns)	0.2064 (ns)
PC ae C38:1	0.5018 (ns)	0.0171 (*)
PC ae C38:2	0.2383 (ns)	0.0364 (*)
PC ae C38:3	0.9599 (ns)	0.1038 (ns)
PC ae C38:4	0.9848 (ns)	0.4641 (ns)
PC ae C38:5	0.9522 (ns)	0.7329 (ns)
PC ae C38:6	0.7581 (ns)	0.7327 (ns)
PC ae C40:0	0.3868 (ns)	0.5049 (ns)
PC ae C40:1 / PC aa C40:8	0.0826 (ns)	0.0428 (*)
PC ae C40:2	0.3745 (ns)	0.2947 (ns)
PC ae C40:3 / PC aa C40:10	0.6400 (ns)	0.6363 (ns)
PC ae C40:4	0.4631 (ns)	0.0308 (*)
PC ae C40:5	0.7081 (ns)	0.6153 (ns)
PC ae C40:6	0.7869 (ns)	0.8658 (ns)
PC ae C42:0	0.5403 (ns)	0.1701 (ns)
PC ae C42:1	0.4149 (ns)	0.1885 (ns)
PC ae C42:2	0.3846 (ns)	0.3106 (ns)
PC ae C42:3	0.9409 (ns)	0.4912 (ns)
PC ae C42:4	0.4634 (ns)	0.1272 (ns)
PC ae C42:5	0.4181 (ns)	0.3050 (ns)
PC ae C44:3	0.7644 (ns)	0.5665 (ns)
PC ae C44:4	0.2343 (ns)	0.1951 (ns)
PC ae C44:5 / PC aa C44:12	0.7296 (ns)	0.1410 (ns)
PC ae C44:6	0.4510 (ns)	0.0939 (ns)

Species	wt 0.25 µM CBD, 72 h<br>p (Welch's t-test)	APPswe 1 µM CBD, 72 h<br>p (Welch's t-test)
PC aa C20:0	0.8312 (ns)	0.2996 (ns)
PC aa C24:0	0.7458 (ns)	0.2179 (ns)
PC aa C26:0	0.5474 (ns)	0.0656 (ns)
PC aa C28:0	0.8198 (ns)	0.0161 (*)
PC aa C28:1	0.8927 (ns)	0.0665 (ns)
PC aa C30:0	0.6702 (ns)	0.0385 (*)
PC aa C30:2	0.9980 (ns)	0.1686 (ns)
PC aa C32:0	0.7235 (ns)	0.9549 (ns)
PC aa C32:1	0.3121 (ns)	0.0091 (**)
PC aa C32:2	0.3239 (ns)	0.0240 (*)

Species	wt 0.25 µM CBD, 72 h<br>p (Welch's t-test)	APPswe 1 µM CBD, 72 h<br>p (Welch's t-test)
PC aa C32:3	0.6422 (ns)	0.1016 (ns)
PC aa C34:1	0.2840 (ns)	0.0245 (*)
PC aa C34:2	0.3860 (ns)	0.0438 (*)
PC aa C34:3	0.5332 (ns)	0.2377 (ns)
PC aa C34:4	0.8325 (ns)	0.0579 (ns)
PC aa C36:0	0.1306 (ns)	<0.0001 (***)
PC aa C36:1	0.0835 (ns)	<0.0001 (***)
PC aa C36:2	0.2507 (ns)	0.0279 (*)
PC aa C36:3	0.2527 (ns)	0.0204 (*)
PC aa C36:4	0.3094 (ns)	0.0265 (*)
PC aa C36:5	0.4036 (ns)	0.1127 (ns)
PC aa C36:6	0.3762 (ns)	0.0438 (*)
PC aa C38:0	0.7103 (ns)	0.3139 (ns)
PC aa C38:1	0.3552 (ns)	0.0252 (*)
PC aa C38:3	0.1909 (ns)	0.0024 (**)
PC aa C38:4	0.5326 (ns)	0.0215 (*)
PC aa C38:5	0.5970 (ns)	0.1693 (ns)
PC aa C38:6	0.4694 (ns)	0.1720 (ns)
PC aa C40:0	0.5000 (ns)	0.4444 (ns)
PC aa C40:1	0.8417 (ns)	0.9231 (ns)
PC aa C40:2	0.6756 (ns)	0.0464 (*)
PC aa C40:3	0.4206 (ns)	0.0337 (*)
PC aa C40:4	0.5801 (ns)	0.0969 (ns)
PC aa C40:5	0.3560 (ns)	0.1576 (ns)
PC aa C40:6	0.2342 (ns)	0.0308 (*)
PC aa C42:0	0.3295 (ns)	0.4207 (ns)
PC aa C42:1	0.4111 (ns)	0.3166 (ns)
PC aa C42:2	0.5490 (ns)	0.2154 (ns)
PC aa C42:4	0.6654 (ns)	0.1318 (ns)
PC aa C42:5	0.3654 (ns)	0.2204 (ns)
PC aa C42:6	0.6941 (ns)	0.1007 (ns)
PC aa C44:0	0.3342 (ns)	0.4173 (ns)
PC aa C48:0	0.4907 (ns)	0.4239 (ns)
PC ae C30:0	0.3203 (ns)	0.0310 (*)
PC ae C30:1	0.5766 (ns)	0.0039 (**)
PC ae C30:2	0.9574 (ns)	0.1520 (ns)
PC ae C32:1	0.1597 (ns)	0.0012 (**)
PC ae C32:2	0.5926 (ns)	0.0303 (*)
PC ae C34:0	0.0268 (*)	0.0473 (*)
PC ae C34:1	0.0554 (ns)	0.0150 (*)
PC ae C34:2	0.2640 (ns)	0.0107 (*)
PC ae C34:3	0.8315 (ns)	0.0257 (*)
PC ae C36:0	0.8691 (ns)	0.0132 (*)
PC ae C36:1	0.3025 (ns)	0.0062 (**)
PC ae C36:2	0.6956 (ns)	0.0443 (*)
PC ae C36:3	0.8265 (ns)	0.1506 (ns)
PC ae C36:4	0.5081 (ns)	0.8755 (ns)
PC ae C36:5	0.9355 (ns)	0.4456 (ns)
PC ae C38:0	0.9023 (ns)	0.0004 (***)
PC ae C38:1	0.0780 (ns)	0.0341 (*)
PC ae C38:2	0.5045 (ns)	0.0873 (ns)
PC ae C38:3	0.9398 (ns)	0.9308 (ns)
PC ae C38:4	0.4556 (ns)	0.4146 (ns)
PC ae C38:5	0.2781 (ns)	0.4963 (ns)
PC ae C38:6	0.5927 (ns)	0.8265 (ns)
PC ae C40:0	0.6175 (ns)	0.0339 (*)
PC ae C40:1 / PC aa C40:8	0.2814 (ns)	0.0510 (ns)
PC ae C40:2	0.4237 (ns)	0.0760 (ns)
PC ae C40:3 / PC aa C40:10	0.4228 (ns)	0.7858 (ns)
PC ae C40:4	0.0932 (ns)	0.4103 (ns)

Species	wt 0.25 µM CBD, 72 h<br>p (Welch's t-test)	APPswe 1 µM CBD, 72 h<br>p (Welch's t-test)
PC ae C40:5	0.0591 (ns)	0.6594 (ns)
PC ae C40:6	0.3041 (ns)	0.7614 (ns)
PC ae C42:0	0.8367 (ns)	0.1433 (ns)
PC ae C42:1	0.3619 (ns)	0.0482 (*)
PC ae C42:2	0.5411 (ns)	0.1766 (ns)
PC ae C42:3	0.9362 (ns)	0.8718 (ns)
PC ae C42:4	0.5190 (ns)	0.6241 (ns)
PC ae C42:5	0.4511 (ns)	0.4441 (ns)
PC ae C44:3	0.1239 (ns)	0.0962 (ns)
PC ae C44:4	0.5591 (ns)	0.0494 (*)
PC ae C44:5 / PC aa C44:12	0.5032 (ns)	0.2206 (ns)
PC ae C44:6	0.5915 (ns)	0.3451 (ns)

Table S8. Number of biological replicates and culture runs for all reported lipidomic experiments.
The number of biological replicates was defined as n and denotes independently incubated, extracted, and measured cell-culture dishes; samples were not pooled or mixed in any way. Only the samples included in the presented results are listed, not all the samples incubated as part of this study. The number in parentheses represents the number of culture runs (all incubated in P3 after thawing). Vertical lines indicate the experimental grouping of certain control and treatment conditions. The three technical mass-spectrometric measurements of each extract were averaged before statistical analysis to one biological value per cell-culture dish and did not raise the n-count artificially.

	24h			72h		72h		72h APPswe	
Lipid class	Control	CBD 0,25µM	CBD 1µM	Control	CBD 0,25µM	Control	CBD 1µM	Control	CBD 1µM
PCaa	11 (1)	12 (1)	13 (1)	14 (1)	11 (1)	36 (3)	19 (3)	12 (1)	13 (1)
PCae	12 (1)	12 (1)	12 (1)	10 (1)	10 (1)	54 (4)	27 (4)	13 (1)	12 (1)
LPC						44 (3)	22 (3)		
SM						36 (3)	19 (3)		
Car						46 (3)	23 (3)		

Table S9. Complete results for the 1 µM/72 h dataset without lipid-class-specific sample exclusion.
The n-values without sample exclusion are (control/CBD): PCaa 46/25, PCae 65/34, LPC 46/25, SM 46/25, Car 46/25.
The n-values with sample exclusion are listed in supplementary table S8. All p-values are Welch's t-test p-values.

	without sample exclusion		with sample exclusion	
Species	Fold change (%)	p-value	Fold change (%)	p-value
PC aa C20:0	101.3	0.827	104.1	0.424
PC aa C24:0	95.7	0.703	98.8	0.919
PC aa C26:0	93.8	0.592	96.6	0.801
PC aa C28:0	105.5	0.497	111.7	0.062
PC aa C28:1	99.7	0.967	103.4	0.641
PC aa C30:0	105.1	0.574	110.6	0.121
PC aa C30:2	86.7	0.090	90.1	0.042
PC aa C32:0	102.5	0.779	106.8	0.286
PC aa C32:1	115.1	0.118	120.4	0.006
PC aa C32:2	95.4	0.583	99.7	0.964
PC aa C32:3	74.1	0.001	77.4	0.000
PC aa C34:1	120.9	0.034	126.2	0.001
PC aa C34:2	110.6	0.249	115.5	0.022
PC aa C34:3	92.3	0.347	95.9	0.456
PC aa C34:4	91.4	0.268	95.2	0.367
PC aa C36:0	154.4	0.000	162.1	0.000
PC aa C36:1	152.2	0.000	159.8	0.000
PC aa C36:2	121.4	0.031	126.8	0.000
PC aa C36:3	107.4	0.424	111.3	0.078
PC aa C36:4	98.4	0.856	102.5	0.685

PC aa C36:5	89.6	0.169	95.0	0.353
PC aa C36:6	100.7	0.929	106.8	0.219
PC aa C38:0	83.3	0.026	87.7	0.026
PC aa C38:1	130.4	0.005	136.2	0.000
PC aa C38:3	119.0	0.055	123.7	0.002
PC aa C38:4	105.1	0.567	109.1	0.158
PC aa C38:5	97.4	0.738	102.0	0.717
PC aa C38:6	98.1	0.822	103.4	0.594
PC aa C40:0	80.4	0.006	84.6	0.002
PC aa C40:1	93.5	0.374	97.2	0.550
PC aa C40:2	120.4	0.033	124.8	0.001
PC aa C40:3	111.4	0.206	114.2	0.028
PC aa C40:4	98.8	0.889	101.4	0.811
PC aa C40:5	101.0	0.902	104.3	0.450
PC aa C40:6	104.5	0.579	109.6	0.112
PC aa C42:0	75.1	0.000	80.3	0.000
PC aa C42:1	68.3	0.000	72.9	0.000
PC aa C42:2	97.8	0.753	103.7	0.438
PC aa C42:4	105.8	0.495	108.3	0.123
PC aa C42:5	99.0	0.905	102.7	0.636
PC aa C42:6	94.4	0.462	98.2	0.730
PC aa C44:0	88.9	0.159	95.0	0.411
PC aa C48:0	91.0	0.227	93.1	0.298
PC ae C30:0	123.1	0.004	128.3	0.000
PC ae C30:1	115.6	0.028	120.4	0.000
PC ae C30:2	89.8	0.102	93.6	0.196
PC ae C32:1	132.6	0.000	137.7	0.000
PC ae C32:2	103.7	0.601	107.6	0.130
PC ae C34:0	146.2	0.000	151.7	0.000
PC ae C34:1	134.1	0.000	138.9	0.000
PC ae C34:2	123.4	0.005	127.4	0.000
PC ae C34:3	103.6	0.583	107.6	0.099
PC ae C36:0	127.9	0.001	133.1	0.000
PC ae C36:1	132.8	0.000	137.2	0.000
PC ae C36:2	116.7	0.030	121.2	0.000
PC ae C36:3	104.1	0.575	107.7	0.148
PC ae C36:4	94.8	0.436	98.5	0.754
PC ae C36:5	96.5	0.577	100.1	0.978
PC ae C38:0	107.4	0.282	111.3	0.025
PC ae C38:1	125.8	0.003	129.3	0.000
PC ae C38:2	115.8	0.041	118.6	0.002
PC ae C38:3	90.6	0.168	92.5	0.129
PC ae C38:4	86.5	0.038	89.3	0.026
PC ae C38:5	82.1	0.004	85.2	0.001
PC ae C38:6	88.7	0.080	92.2	0.089
PC ae C40:0	95.7	0.502	99.1	0.850
PC ae C40:1 / PC aa C40:8	109.9	0.168	112.9	0.016
PC ae C40:2	109.3	0.204	113.0	0.022
PC ae C40:3 / PC aa C40:10	90.2	0.136	93.0	0.154
PC ae C40:4	73.7	0.000	76.5	0.000
PC ae C40:5	74.9	0.000	77.1	0.000
PC ae C40:6	83.2	0.007	86.6	0.005
PC ae C42:0	94.3	0.334	98.4	0.697
PC ae C42:1	89.6	0.063	92.0	0.030
PC ae C42:2	93.6	0.281	95.7	0.308
PC ae C42:3	93.7	0.306	96.6	0.499
PC ae C42:4	91.5	0.180	94.9	0.281
PC ae C42:5	93.4	0.293	97.8	0.672
PC ae C44:3	83.9	0.006	86.9	0.017
PC ae C44:4	88.9	0.044	91.8	0.060
PC ae C44:5 / PC aa C44:12	94.1	0.324	97.0	0.487
PC ae C44:6	95.2	0.471	99.8	0.979

Lyso PC C06:0	98.7	0.896	97.8	0.829
Lyso PC C10:0	87.3	0.276	80.6	0.015
Lyso PC C12:0	87.4	0.205	90.3	0.326
Lyso PC C14:0	82.0	0.002	79.4	0.000
Lyso PC C16:0	87.9	0.036	85.8	0.016
Lyso PC C16:1	80.0	0.001	78.2	0.001
Lyso PC C17:0	88.0	0.007	87.4	0.004
Lyso PC C18:0	109.0	0.116	106.7	0.229
Lyso PC C18:1	87.7	0.027	85.8	0.014
Lyso PC C18:2	80.4	0.001	78.7	0.000
Lyso PC C18:3	83.0	0.002	82.0	0.001
Lyso PC C20:0	82.4	0.028	82.1	0.014
Lyso PC C20:3	74.0	0.000	72.7	0.000
Lyso PC C20:4	67.0	0.000	66.5	0.000
Lyso PC C20:5	87.8	0.058	87.4	0.025
Lyso PC C22:0	65.6	0.004	65.4	0.003
Lyso PC C22:6	72.3	0.000	70.9	0.000
Lyso PC C24:0	77.5	0.054	76.9	0.046
Lyso PC C26:0	84.5	0.168	85.4	0.187
Lyso PC C26:1	80.7	0.030	80.7	0.024
Lyso PC C28:0	98.7	0.875	98.1	0.812
Lyso PC C28:1	80.7	0.060	80.5	0.051
SM C14:1 OH	88.5	0.041	95.9	0.350
SM C16:0	81.5	0.001	86.3	0.003
SM C16:1	88.6	0.057	94.4	0.223
SM C16:1 OH	83.1	0.003	88.1	0.007
SM C18:0	78.3	0.000	82.3	0.000
SM C18:1	67.7	0.000	71.7	0.000
SM C20:2	72.9	0.000	76.7	0.000
SM C22:1 OH	97.3	0.636	101.5	0.730
SM C22:2 OH	71.7	0.000	74.2	0.000
SM C24:0	118.6	0.004	124.8	0.000
SM C24:1	98.0	0.727	102.5	0.584
SM C24:1 OH	94.0	0.280	97.8	0.599
SM C26:0	100.9	0.874	105.1	0.247
SM C26:1	90.9	0.098	94.0	0.156
C0	79.2	0.000	78.4	0.000
C02	73.3	0.000	72.0	0.000
C03	70.6	0.000	69.3	0.000
C03 OH	75.1	0.000	74.8	0.000
C03:1	82.2	0.001	82.6	0.001
C04	72.1	0.000	72.4	0.000
C04 OH / C3 DC	83.2	0.003	83.2	0.001
C04:1	90.7	0.011	90.7	0.009
C05	67.2	0.000	66.1	0.000
C05 M DC	99.7	0.895	100.2	0.920
C05 OH / C3 M DC	77.4	0.000	76.7	0.000
C05:1	88.7	0.045	88.0	0.009
C05:1 DC	80.1	0.001	80.4	0.001
C06 / C4:1 DC	83.4	0.003	84.3	0.003
C06 OH / C5 DC	96.5	0.612	96.6	0.559
C06:1	69.3	0.000	69.5	0.000
C07 DC	78.6	0.000	77.7	0.000
C08	93.0	0.525	89.2	0.312
C08:1	84.9	0.001	85.4	0.000
C09	85.3	0.011	84.4	0.003
C10	88.9	0.015	89.3	0.009
C10:1	72.5	0.003	73.2	0.005
C10:2	91.2	0.165	93.3	0.291
C12	79.3	0.001	76.8	0.000
C12 DC	92.7	0.296	91.4	0.200
C12:1	80.9	0.001	81.7	0.002

C14	76.3	0.001	76.2	0.000
C14:1	82.5	0.004	84.8	0.008
C14:1 OH	87.4	0.056	88.4	0.068
C14:2	90.9	0.081	91.2	0.067
C14:2 OH	82.4	0.008	81.9	0.005
C16	99.5	0.966	96.2	0.675
C16 OH	95.7	0.555	95.9	0.557
C16:1	70.8	0.000	72.6	0.000
C16:1 OH	86.0	0.011	88.1	0.031
C16:2	87.1	0.021	86.0	0.005
C16:2 OH	93.2	0.260	95.7	0.477
C18:0	89.5	0.218	88.4	0.142
C18:1	62.3	0.000	63.8	0.000
C18:1 OH	109.8	0.163	110.4	0.131
C18:2	78.7	0.000	80.9	0.001
